# Structural and Functional Insights Into Lysostaphin–Substrate Interaction

**DOI:** 10.3389/fmolb.2018.00060

**Published:** 2018-07-03

**Authors:** Helena Tossavainen, Vytas Raulinaitis, Linda Kauppinen, Ulla Pentikäinen, Hannu Maaheimo, Perttu Permi

**Affiliations:** ^1^Department of Chemistry, Nanoscience Center, University of Jyvaskyla, Jyvaskyla, Finland; ^2^Program in Structural Biology and Biophysics, Institute of Biotechnology, University of Helsinki, Helsinki, Finland; ^3^Department of Biological and Environmental Science, University of Jyvaskyla, Jyvaskyla, Finland; ^4^Institute of Biomedicine, University of Turku, Turku, Finland; ^5^Turku Centre for Biotechnology, Turku, Finland; ^6^VTT Technical Research Centre of Finland Ltd., Espoo, Finland

**Keywords:** lysostaphin, NMR structure, pentaglycine, peptidoglycan, protein dynamics, SH3b domain, *Staphylococcus aureus*, substrate binding

## Abstract

Lysostaphin from *Staphylococcus simulans* and its family enzymes rapidly acquire prominence as the next generation agents in treatment of *S. aureus* infections. The specificity of lysostaphin is promoted by its C-terminal cell wall targeting domain selectivity toward pentaglycine bridges in *S. aureus* cell wall. Scission of these cross-links is carried out by its N-terminal catalytic domain, a zinc-dependent endopeptidase. Understanding the determinants affecting the efficiency of catalysis and strength and specificity of interactions lies at the heart of all lysostaphin family enzyme applications. To this end, we have used NMR, SAXS and molecular dynamics simulations to characterize lysostaphin structure and dynamics, to address the inter-domain interaction, the enzyme-substrate interaction as well as the catalytic properties of pentaglycine cleavage in solution. Our NMR structure confirms the recent crystal structure, yet, together with the molecular dynamics simulations, emphasizes the dynamic nature of the loops embracing the catalytic site. We found no evidence for inter-domain interaction, but, interestingly, the SAXS data delineate two preferred conformation subpopulations. Catalytic H329 and H360 were observed to bind a second zinc ion, which reduces lysostaphin pentaglycine cleaving activity. Binding of pentaglycine or its lysine derivatives to the targeting domain was found to be of very low affinity. The pentaglycine interaction site was located to the N-terminal groove of the domain. Notably, the targeting domain binds the peptidoglycan stem peptide Ala-d-γ-Glu-Lys-d-Ala-d-Ala with a much higher, micromolar affinity. Binding site mapping reveals two interaction sites of different affinities on the surface of the domain for this peptide.

## Introduction

The gram-positive pathogen *Staphylococcus aureus* causes numerous diseases, ranging from minor skin abscesses to meningitis and even toxic shock syndrome (Lowy, 1998). *S. aureus* bacteremia is estimated to be fatal in up to 30 % of cases (van Hal et al., 2012) and annually incur billions in overall medical costs just in the US (Lee et al., 2013) and hundreds of millions of additional hospitalization costs in the EU (Köck et al., 2010). The problem is exacerbated by the pathogen being carried by one third of human population (Chambers and Deleo, 2009) and its vigor to develop resistance against drugs mobilized for its treatment. Hence, hospital-acquired *S. aureus* cases are particularly severe.

For seven decades treatment of *S. aureus* infections primarily relied on antibiotics interfering with the synthesis of its cell wall, specifically, the penicillin methicillin and the glycopeptide vancomycin. Inhibitors of protein synthesis have been recently added to the arsenal of therapeutic means (Brickner et al., 2008). However, the historic propensity of bacteria to rapidly develop resistance to small molecules impels the conception of new treatment paradigms (Taubes, 2008). Today *S. aureus* appears as one of the 12 most health-threatening pathogens in the list of resistant bacteria compiled by WHO to promote development of new antibiotics (WHO Mediacentre, 2017).

Pentaglycine cross-bridges of peptidoglycan (PG) are a distinct feature of the *S. aureus* cell wall and provide innate selectivity as targets (Vollmer et al., 2008). Some members of the *Staphylococcus* genus have taken advantage of this feature and secrete bactericidal enzymes which cleave this cross-bridge to lyse competing *S. aureus* (Schindler and Schuhardt, 1964; Browder et al., 1965; Sugai et al., 1997), with their own intrinsic resistance engineered by serine replacements of the glycine residues (Thumm and Götz, 1997; Tschierske et al., 1997). Scission of pentaglycine is executed by endopeptidases of the lysostaphin family, which also includes *S. aureus* inherent autolytic enzymes that maintain the integrity of the cell wall during cell life cycle (Ramadurai et al., 1999; Ercoli et al., 2015; Raulinaitis et al., 2017a).

A modular organization is common to many peptidoglycan hydrolases (Szweda et al., 2012). Mature lysostaphin consists of an N-terminal catalytic (CAT) domain, and a C-terminal cell wall targeting (CWT) domain (Baba and Schneewind, 1996). Its X-ray crystal structure has been reported recently (Sabala et al., 2014). The CAT domain forms a barrel fold typical for the MEROPS M23 family zinc-dependent endopeptidases, which bear conserved catalytic as well as Zn^2+^-coordinating amino acid residues (Rawlings et al., 2008). A fourteen-residue linker connects the CAT domain to the CWT domain. The latter assumes the fold corresponding to SH3b domains (Lu et al., 2006). The SH3b domains are the bacterial counterpart of the ubiquitous SH3 domains found in eukaryotes (Ponting et al., 1999; Whisstock and Lesk, 1999). These are small interaction modules present in proteins of signaling pathways, which interact with proline-rich sequences in binding partners (Saksela and Permi, 2012). The canonical PXXP binding site on the surface of SH3 domains is partially blocked in the SH3b domains, and the pentaglycine interaction site of SH3b domains has been located to a groove structure formed by a 20-residue N-terminal extension not present in the smaller SH3 domains (Lu et al., 2006; Hirakawa et al., 2009; Gu et al., 2014). Despite the linker, evidence for CAT–CWT domain interaction *in vitro* has been presented, although the same study underscored the benefits of flexibility between the domains *in vivo* (Lu et al., 2013).

Surprisingly little is known about the details of the interaction between lysostaphin and PG. Biochemical characterization has revealed that the CWT domain is indispensable for lysostaphin specificity, and that an intact pentaglycine cross-bridge is crucial for the CWT–PG interaction (Gründling and Schneewind, 2006). The interaction can be inhibited by PG fragments containing multiple cross-linked murein subunits, but not with smaller murein mono- or dimers. Lipoproteins, cell wall-anchored proteins and wall teichoic acids appear not to have a role in the interaction (Gründling and Schneewind, 2006). Similarly, PG binding of *Staphylococcus capitis* ALE-1 CWT domain, which is highly similar in sequence and structure to the lysostaphin CWT domain, is dependent on the length and amino acid composition of the cross-bridge, showing strong preference for Gly_5_ bridges (Lu et al., 2006). The complex structure of lysostaphin CWT domain with pentaglycine peptide is a landmark in the characterization of the SH3b–PG interaction (PDB ID 5LEO). In this structure pentaglycine resides in the N-terminal groove, consistent with previous mutational, computational and NMR studies.

Medical applications of recombinant lysostaphin are advancing and some have entered clinical trials (Nelson et al., 2012). Lysostaphin CWT domain fusion proteins have also been considered as a promising source of alternative chemotherapeutics (Schmelcher et al., 2012; Osipovitch and Griswold, 2015; Jagielska et al., 2016). In addition, lysostaphin has been engineered to suppress its immunogenic potential (Blazanovic et al., 2015). It is clear that the key factor in the success of all lysostaphin family enzyme applications is understanding the determinants affecting efficiency of catalysis and strength and specificity of interactions. To this end, we have used NMR, SAXS and molecular dynamics (MD) simulations to characterize lysostaphin structure and dynamics, to address the inter-domain interaction, the enzyme-substrate interaction as well as the catalytic properties of pentaglycine cleavage in solution.

## Materials and methods

### Production and purification of lysostaphin and its CWT domain

Gene for mature lysostaphin (residues 251-493) was synthesized de novo at GenScript (NJ, USA) and cloned into pGEX-2T vector (GE Healthcare Life Sciences) at the BamH1 and EcoR1 cloning sites. Resulting plasmid was transformed into *E. coli* BL21 (DE3) strain. DNA encoding the CWT domain (residues 402-493) was amplified (primers 5′-CGCGGATCCTGGAAAACCAATAAATATGGCACGC-3′ and 3′-CGCGAATTCTTATTTGA TGGTGCCCCACAGG-5′) using this plasmid as template and was cloned into pGEX-2T vector separately at the BamH1 and EcoR1 cloning sites as well.

Protein expression and purification was carried out as described in detail previously (Raulinaitis et al., 2017a). Overnight cell cultures were inoculated into a fresh medium. LB medium was used for non-labeled protein production and when labeled protein was required for NMR data collection, cells were grown in minimal resource M9 medium (40 mM Na_2_HPO_4_, 22 mM KH_2_PO_4_, 12 mM NaCl, 1 mM MgSO_4_, 100 μM CaCl_2_, 10 μM FeSO_4_, 0.001 % w/w biotin, and 0.1 mg/ml ampicillin, pH 7.0). Upon cells reaching OD600, protein expression was induced with isopropyl β-D-1-thiogalactopyranoside (IPTG), 0.5 mM final concentration, and further incubated for 3 h. Harvested cells were disrupted by sonication, and soluble fraction was loaded on Glutathione Sepharose 4 Fast Flow resin (GE Healthcare). Lysostaphin was released from resin-bound glutathione tag by thrombin and protein was further purified by gel filtration on HiLoad 16/60 Superdex 75 column (GE Healthcare Life Sciences). Removal of innately bound metal ions was carried out with EDTA, which was thereafter dialyzed away in PBS buffer and protein was concentrated with Amicon^®;^ 5000 MWCO centrifugal filters.

### NMR spectroscopy

Spectra for chemical shift assignment and structure determination were acquired at 308 K on a Varian INOVA 800 MHz spectrometer equipped with a cryogenically cooled triple resonance probehead equipped with an actively shielded z-gradient. Resonance assignment was carried out with the following set of experiments: ^1^H, ^15^N HSQC, ^1^H, ^13^C HSQC, ^1^H, ^13^C CT-HSQC, HNCACB, CBCA(CO)NH, (H)CC(CO)NH, H(CCO)NH, HBHA(CO)NH, (HB)CB(CGCD)HD, (HB)CB(CGCDCE)HE, ^1^H, ^15^N NOESY-HSQC, ^1^H, ^13^C NOESY-HSQC in 7% D_2_O/93% H_2_O, and ^1^H, ^13^C HSQC-NOESY in 100% D_2_O. The latter five spectra were employed for the assignment of the aromatic residues as well as the source of distance restraints for structure determination. Additional structural restraints were derived from the chemical shifts in the form of TALOS-N (Shen and Bax, 2013) dihedral angle restraints and applied for residues in secondary structures as well as H_2_O to D_2_O exchange experiment in the form of hydrogen bond restraints for amides experiencing slow exchange. Histidine tautomeric state could be derived from the peak pattern in a ^1^H, ^15^N HMBC spectrum or the Cδ2-Cε1 chemical shift difference for four out of nine histidines (Barraud et al., 2012). Spectra were processed with NMRPipe (Delaglio et al., 1995) and analyzed with Sparky (T. D. Goddard and D. G. Kneller, University of California, San Francisco). The automated NOE peak assignment-structure determination routine of CYANA 2.1 (López-Méndez and Güntert, 2006) was used to generate an ensemble of 20 lowest target energy structures which were further refined with AMBER 16 (Case et al., 2005) in explicit solvent. Fifteen lowest AMBER energy structures were chosen to represent the solution state structure of lysostaphin.

The pentaglycine binding site on the CWT domain was determined by comparing the ^1^H, ^15^N, and ^1^H, ^13^C HSQC spectra of full-length lysostaphin and CWT domain acquired in the presence and absence of a large amount, ~170 molar excess, of KGGGGG and GGGGGK hexapeptides (Sigma-Aldrich). Binding characteristics of a PG peptide mimic were determined by titration of the CWT domain with A-d-EKGGGGGA-d-EK-d-A and comparing ^1^H, ^15^N HSQC spectra acquired at approximate peptide to CWT domain molar ratios of 0, 0.5, 1, 2, 5, 10, and 24. Similarly, the pentapeptide A-d-γ-EK-d-A-d-A (Bachem) and muramyl dipeptide *N*-acetylmuramyl-l-alanyl-d-isoglutamine hydrate (Carbosynth Ltd) were titrated to a CWT domain sample, and ^1^H, ^15^N HSQC spectra were collected at approximate peptide to CWT domain molar ratios of 0, 0.5, 1, 2, 4, 8, 16, 32, 45, and 60. CSPs, calculated as Δδ = (ΔδH^2^+(0.154 × ΔδN)^2^)^1/2^, were plotted as a function of the molar ratio of titrated ligand to CWT domain. Dissociation constants for individual residues were obtained by nonlinear least squares fitting, as implemented in the program xcrvfit (www.bionmr.ualberta.ca/bds/software/xcrvfit). Site-specific dissociation constants were calculated as averages of dissociation constants of individual residues within the site. In all binding site studies protein to zinc concentration ratio was 1:1.

CAT domain–SH3 domain interaction was probed by replacing zinc with paramagnetic manganese (1:1 protein to cation ratio) and observing peak bleaching in a ^1^H, ^15^N HSQC spectrum.

^15^N T_1_, T_2_ and heteronuclear NOE spectra were acquired for one- and three-zinc bound lysostaphin in the presence and absence of substrate pentaglycine on a Bruker AVANCE III HD 800 MHz spectrometer, equipped with a TCI ^1^H/^13^C/^15^N cryoprobe. For T_1_ relaxation delays of 20, 60, 100, 200, 400, 600, 900, 1,400, 2,000, and 2,600 ms were used. For T_2_ the delays were multiples of loop length 16.96 ms, with the loop counter set to 1, 2, 3, 4, 5, 6, 7, 8, 10, and 12. The recycle delay was set to 3.1 and 2.6 s for the T_1_ and T_2_ experiments, respectively. All spectra were acquired as pseudo 3D spectra and Fourier transformed with NMRPipe. Peak intensities were fitted to decaying exponential function as implemented in Sparky. Heteronuclear NOE values were calculated as the intensity ratios of peaks from a pair of spectra measured with and without ^1^H presaturation during the recycle delay. The relaxation delay was set to 5 s. The CWT domain relaxation spectra were recorded on a Bruker AVANCE III 500 MHz spectrometer. For T_1_, relaxation delays of 20, 60, 100, 200, 400, 600, 800, 1,200, 1,600, and 2,000 ms. The recycle delay was set to 2.5. For T_2_, the loop counter was set to 1, 2, 4, 8, 10, 12, 14, and 16 and the recycle delay was 1.5 s. Heteronuclear NOE spectra were acquired as for full-length lysostaphin.

Pentaglycine cleavage by lysostaphin was monitored by ^1^H NMR as described previously (Raulinaitis et al., 2017a). Reactions took place in phosphate buffer saline (PBS), pH 7.2, with >90% D_2_O by volume as a solvent and final volumes were 600 μL. Initial pentaglycine (Sigma-Aldrich) and lysostaphin concentrations were 1 mM and 2.25 μM, respectively. Metal ions (ZnCl_2_, CoCl_2_, CuCl_2_, and MnCl_2_) were added as needed. Residual activity of lysostaphin “apo” form was taken into account in activity calculations. Measurements were carried out by using a Bruker Avance III 600 MHz spectrometer, equipped with a QCI ^1^H/^13^C/^15^N/^31^P cryoprobe and a SampleJet automated sample changer. The preheater of the sample changer was used for sample incubation when product formation was measured as a time series with 3-h intervals for 48 h. Single time-point samples were incubated in an incubator for 21 h and reactions were quenched by heating at 90°C for 15 min before proton spectra acquisitions at 37°C. The data were processed with Bruker TopSpin 3.5 software.

### MD simulations

MD simulations in explicit solvent were performed with AMBER 16 (Case et al., 2005) using the ff14SB force field. Lysostaphin CYANA ensemble structures 1, 5, 7, and 14 were selected as targets of simulations. The topology and coordinate files were generated with the LEaP program in AMBER 16. The zinc ion was modeled using the cationic dummy atom approach (Pang, 1999; Pang et al., 2000). The protein molecule was placed in a cubic box with a minimum solute-box distance of 10 Å, and solvated with TIP3P water molecules. Nine chloride ions were added to maintain electrical neutrality. After minimization, heating and equilibration of the system, the production 30-ns MD simulations were performed with periodic boundary conditions at 300 K. The temperature was maintained by using the Langevin thermostat, whereas the pressure was kept at 1 bar using the Berendsen barostat (Berendsen et al., 1984). The time step was set to 2 fs. Long-range electrostatic interactions were treated using the Particle Mesh Ewald method (Darden et al., 1993) with a cut-off of 10 Å. Bond lengths involving hydrogen atoms were constrained by SHAKE (Ryckaert et al., 1997). Analyses of the trajectories were carried out with CPPTRAJ (Roe and Cheatham, 2013) and VMD (Humphrey et al., 1996).

### SAXS

BM29 beamline, ESRF, Grenoble, France was used for collecting the SAXS data on a PILATUS 1M image plate using sample to detector distance of 2.9 m and a wavelength of 1.0 Å (momentum transfer range 0.01 < q < 5 nm^−1^). The measurements were carried out at 288 K in PBS buffer. Three different protein concentrations (1.0, 3.0, and 5.0 mg/ml) were used in the data acquisition. PRIMUS (Konarev et al., 2003) in ATSAS software package (Franke et al., 2017) was utilized in the data processing. To assess the conformational variability of lysostaphin, EOM (Tria et al., 2015) was used. In EOM calculations, lysostaphin CAT domain (residues 251-384) and CWT domain (residues 410-493) of the NMR structure reported here, were used as rigid bodies, and residues 385-409 as random coil. 10 000 randomized conformations were generated for lysostaphin based on amino acid sequence and 3D-structure of CAT and CWT domains of lysostaphin using both random coil and native options in EOM. The scattering profiles of these randomly generated conformations were compared, and representative 20 structures whose scattering curve fits to the experimental scattering curve were selected by EOM algorithm. EOM run was repeated 100 times to provide statistical data about lysostaphin radius of gyration (*R*_*g*_) and maximum dimensions (*D*_*max*_) distribution.

## Results and discussion

### Lysostaphin catalytic groove embracing loops are highly mobile

**Figure 1:**
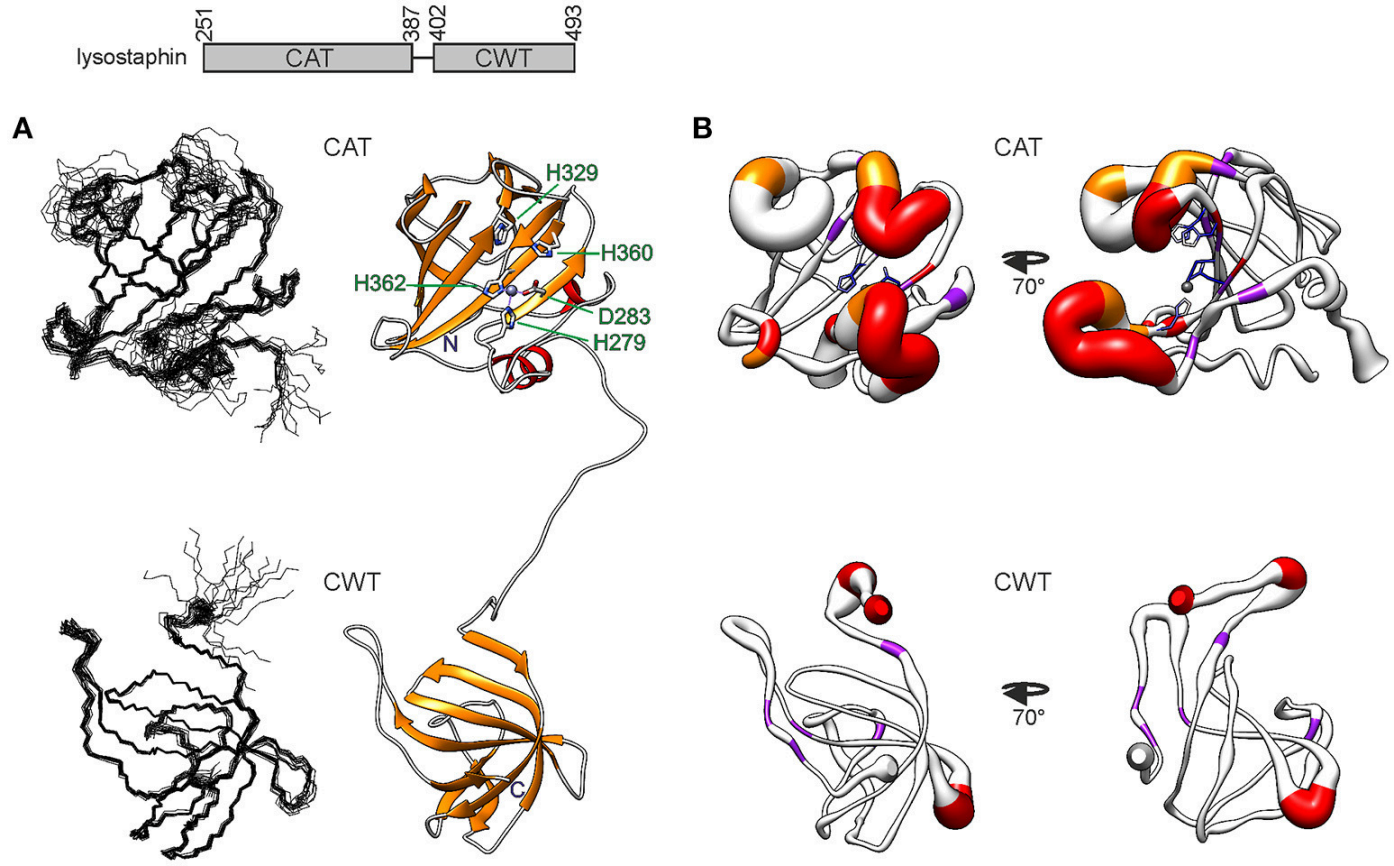
Structure of *S. simulans* lysostaphin. On top, the domain structure is depicted. The N-terminal CAT domain is composed of 137 residues and the C-terminal CWT domain of 92 residues. The domains are linked by a flexible 14-residue linker. **(A)** Solution NMR structure of lysostaphin. On the left, the two domains are represented as ensembles of 15 superimposed structures. On the right, a representative member of the ensemble is shown in a ribbon diagram. The catalytic site residues in the CAT domain are labeled. Zinc is shown as a gray sphere. **(B)** CAT (top) and CWT domain dynamics. The heavy atom RMSF obtained from the MD simulation is depicted as the radius of the ribbon. Residues which lack or display strongly attenuated amide peak in the ^1^H, ^15^N HSQC spectrum are colored in red and orange, respectively. Residues which experience μs-ms dynamics are colored in purple. Catalytic site residues in the CAT domain are colored according to their heavy atom RMSF, blue depicting low RMSF values and lighter colors higher RMSF values. The structure on the left has the same orientation as the structures in **(A)**, whereas the structure on the right has been rotated 70 degrees around the vertical axis.

The solution NMR structure of lysostaphin was determined based on distance restraints from NOESY spectra, φ/ψ dihedral restraints derived from chemical shifts using TALOS-N (Shen and Bax, 2013) and hydrogen bond restraints obtained from a H/D exchange experiment (Figure 1A and Table 1). In line with the X-ray crystal structure (Sabala et al., 2014), lysostaphin is structurally arranged into two domains. The domains are connected by a fourteen-residue linker, which is devoid of persistent structure. The domains have random mutual orientations in the ensemble of structures. The CAT domain shares the structural features of a MEROPS M23 metallopeptidase domain with a β-sheet core and four loops forming the bottom and the rims of the catalytic groove, respectively (Rawlings et al., 2008). In one end of the groove reside the catalytic residues surrounding a zinc cation. The CWT domain has the SH3b domain, barrel-like all β-sheet fold.

**Table 1:** NMR restraints and structural statistics for the ensemble of 15 lysostaphin conformers of least restraint violations tabulated for the whole enzyme and domains separately.

	**Lysostaphin**	**CAT**	**CWT**
**COMPLETENESS OF RESONANCE ASSIGNMENTS (%)**^a^			
Backbone	93.3	92.1	98.3
Side chain, aliphatic	93.7	93.3	97.4
aromatic	95.7	91.7	99.5
**EXPERIMENTAL RESTRAINTS**			
**Distance restraints**
Total	4312	2208	2070
Short range (i-j ≤ 1)	1950	1017	903
Medium range (1 < i-j < 5)	448	245	199
Long range (i-j≥5)	1914	946	968
H-bond restraints	44	30	14
Dihedral restraints (TALOS-N)	277	153	124
No. of restraints per restrained residue	22.9	19.8	24.8
No. of long-range restraints per restrained residue	9.7	8.6	11.0
**RESIDUAL RESTRAINTS VIOLATIONS**			
**Average no. of distance violations per structure**
0.1–0.2 Å	10.9		
0.2–0.5 Å	1.1 (max 0.3 Å)
>0.5 Å	0		
**Average no. of dihedral angle violations per structure**
1–5°	0		
>5°	0		
**MODEL QUALITY**^b^			
RMSD backbone atoms (Å)		0.6	0.3
RMSD heavy atoms (Å)		1.0	0.7
RMSD bond lengths (Å)	0.014		
RMSD bond angles (°)	2.0		
**MOLPROBITY RAMACHANDRAN STATISTICS (%)**^b^			
Most favored regions	97.0	97.0	96.9
Allowed regions	3.0	2.9	3.1
Disallowed regions	0.0	0.1	0.0
**GLOBAL QUALITY SCORES (RAW/Z SCORE)**^b^			
Verify3D	0.44/−0.32	0.43/−0.48	0.46/0.00
ProsaII	0.33/−1.32	0.20/−1.86	0.45/−0.83
PROCHECK(ϕ-ψ)	−0.43/−1.38	−0.40/−1.26	−0.48/−1.57
PROCHECK (all)	−0.40/−2.37	−0.36/−2.13	−0.43/−2.54
Molprobity clash score	1.19/1.32	0.45/1.45	2.30/1.13
**MODEL CONTENTS**			
No. of ordered residues^b^	202	113	89
Total no. of residues	245	139	92
BMRB accession number	34121		
PDB ID code	5NMY		

a *Backbone includes Cα, Cβ, N, and H atoms, except the N-terminal amide. For side chains, excluded are the highly exchangeable groups (Lys, amino, Arg, guanido, Ser/Thr/Tyr hydroxyl, His δ1/ε2), as well as all unprotonated carbons and nitrogens*.

b *Ordered residues: 253–270, 279–308, 313–350, 361–387, 402–465, 468–492. Computed using PSVS (Bhattacharya et al., 2007)*.

**Figure 2:**
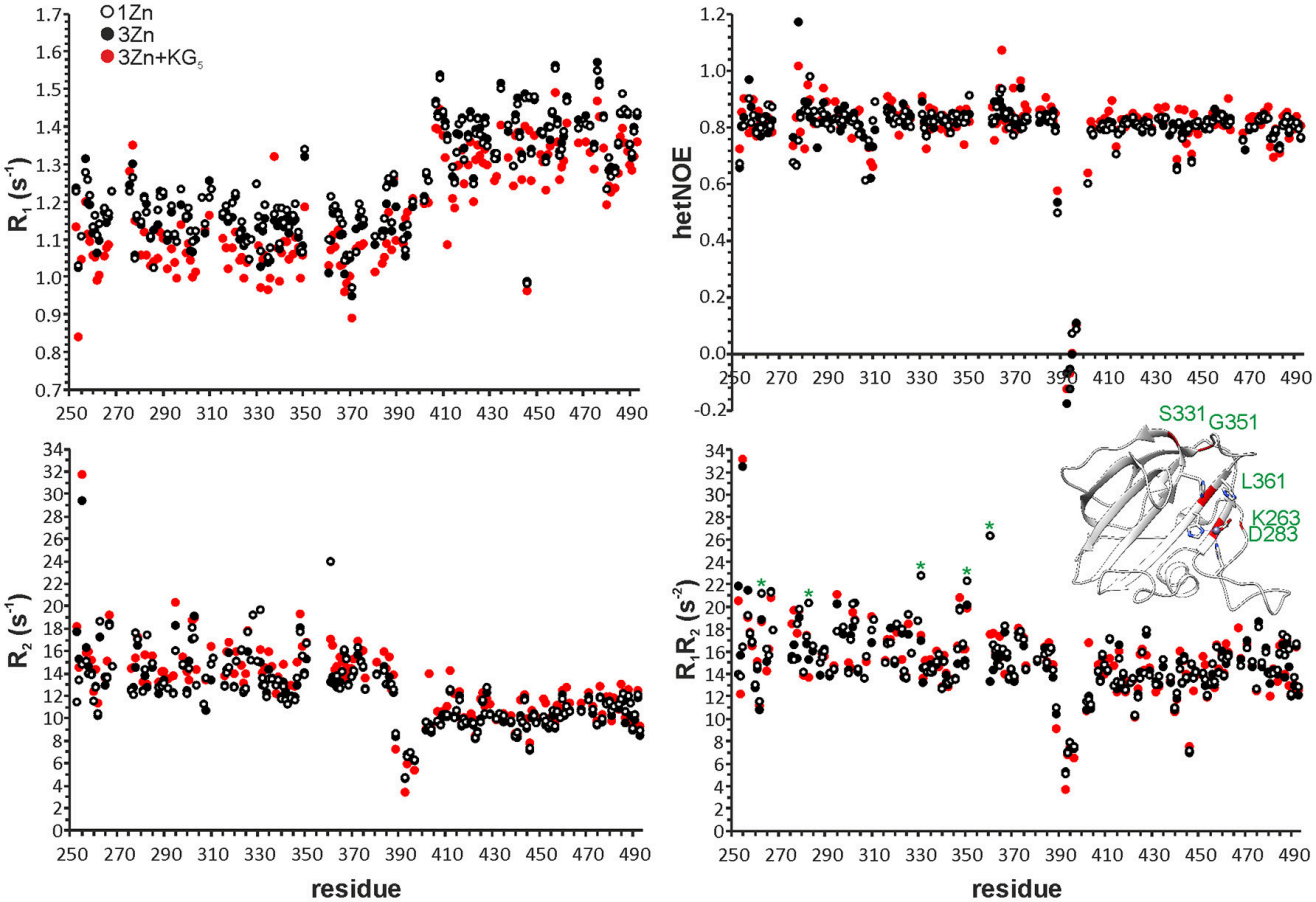
^15^N R_1_, R_2_, heteronuclear NOE and R_1_R_2_ data of one-zinc (white circles), three-zinc (black circles) and three-zinc lysostaphin in the presence of ~140 molar excess of KG_5_ (red circles). Error bars have been omitted for clarity. Average errors are: 1Zn CAT 0.01 s^−1^ (R_1_), 0.19 s^−1^ (R_2_), 0.03 (HetNOE) and 0.29 s^−2^ (R_1_R_2_); 1Zn CWT 0.01 s^−1^ (R_1_), 0.10 s^−1^ (R_2_), 0.01 (HetNOE) and 0.16 s^−2^ (R_1_R_2_); 3Zn CAT 0.01 s^−1^ (R_1_), 0.20 s^−1^ (R_2_), 0.03 (HetNOE) and 0.27 s^−2^ (R_1_R_2_); 3Zn CWT 0.01 s^−1^ (R_1_), 0.11 s^−1^ (R_2_), 0.01 (HetNOE), and 0.16 s^−2^ (R_1_R_2_); 3Zn+KG_5_ CAT 0.02 s^−1^ (R_1_), 0.26 s^−1^ (R_2_), 0.06 (HetNOE) and 0.40 s^−2^ (R_1_R_2_); 3Zn+KG_5_ CWT 0.01 s^−1^ (R_1_), 0.08 s^−1^ (R_2_), 0.04 (HetNOE) and 0.14 s^−2^ (R_1_R_2_). In the R_1_R_2_ panel residues experiencing a large change upon addition of excess zinc are marked with asterisks and highlighted in red in the CAT domain structure.

While the core of the CAT domain is well-defined, and nicely superimposable with the crystal structure (RMSDs of 0.7 and 1.3 Å for backbone and heavy atoms, respectively), the catalytic groove embracing loops and the catalytic histidines are conformationally dispersed, which results in a poorer overall structural match in these regions. The conformational spread in the NMR ensemble is the result of solvent exposure and flexibility of residues in these regions (Figure 1B and Supplementary Figure 1). Fast amide proton exchange with solvent is likely to be present in the loops, because these do not display or have severely broadened peaks in a ^1^H, ^15^N HSQC spectrum. Additionally, the catalytic histidines probably exhibit solvent and/or conformational exchange, because their signals were not observed in a histidine-region ^1^H, ^15^N HMBC spectrum, showing cross-peaks between Nδ1, Nε2, Hδ2, and Hε1. Although carbon-bound Hδ2 or Hε1 do not exchange with solvent, they can be influenced by solvent or conformational exchange elsewhere in the aromatic ring. This manifests as additional line-broadening of histidine ring protons and disappearance of catalytic histidine correlations from the HMBC. In addition to exchange, micro- to milli-second time scale motions are present in the catalytic groove (Figure 2 and Table 2), as identified by high R_1_R_2_ products derived from ^15^N relaxation measurements (Kneller et al., 2002).

**Table 2:** Average R_1_, R_2_ and hetNOE values and standard deviations derived from ^15^N relaxation experiments at 800 MHz, 35°C.

**Cofactor/Ligand**	**Domain**	**R_1_ (s^−1^)**	**R_2_ (s^−1^)**	**hetNOE**	***τ_*c*_* (ns)**
1Zn	CAT	1.15 ± 0.07	13.60 ± 1.49	0.83 ± 0.05	7.9 ± 0.5
	CWT	1.37 ± 0.09	10.10 ± 1.08	0.79 ± 0.06	6.0 ± 0.4
3Zn	CAT	1.13 ± 0.07	13.61 ± 1.51	0.83 ± 0.06	7.9 ± 0.5
	CWT	1.38 ± 0.08	9.86 ± 1.01	0.80 ± 0.04	5.9 ± 0.3
3Zn + KG_5_	CAT	1.06 ± 0.07	14.78 ± 1.69	0.84 ± 0.07	8.6 ± 0.6
	CWT	1.31 ± 0.08	10.59 ± 1.23	0.82 ± 0.07	6.3 ± 0.4

*Residues with significant contribution from internal motions were not included in the calculation of averages (Tjandra, 1996)*.

Catalytic groove conformational dynamics is evident also from MD simulations. The root mean square fluctuation, RMSF, emphasizes the flexible nature of three of the groove embracing loops (Figure 1B and Supplementary Figure 1). Consistent with the NMR ensemble, the outer half of the N-terminal loop, residues G270-G277, is highly dynamic, whereas the inner half is restrained by transient backbone hydrogen bonds within the loop, Y267-G281, G268-H279 as well as the H279Nε2–Zn^2+^ interaction. The loop encompassing residues N368-Q376 is the least flexible. It is transiently stabilized by backbone S371-T374 and side chain S369-T374 hydrogen bonds, the former twisting the loop into a three-residue 3_10_ helix.

The MD simulation reveals several possible mechanisms contributing to the disappearance of the catalytic histidine HMBC signals (Supplementary Figure 1). Firstly, while the two histidine rings remain approximately coplanar during the simulation, they slide relative to each other. Secondly, the aromatic ring of F285, next to H360, is able to rotate. Thirdly, the loop closest to the catalytic histidines, residues T353-Y355 thereof in particular, adopt a multitude of conformations at a fluctuating distance from the catalytic histidines. Conformational exchange between all these different structural arrangements is likely to broaden the HMBC signal beyond detection. Moreover, perhaps most importantly, H329 and H360 are transiently accessible to solvent, leading to signal broadening through exchange between tautomeric, ionic and H/D states. Notably, the outward orientation of H360 aromatic ring in some of the NMR ensemble structures appears to be energetically unfavorable as during the MD simulations the ring turns toward the catalytic groove.

Of the M23 family endopeptidases, for which the structure has been determined, lysostaphin CAT domain has the highest amino acid sequence identity with the sequences of LytM and LytU, 49 and 44%, respectively. LytM and LytU are *S. aureus* autolytic endopeptidases taking part in PG remodeling by cleaving the pentaglycine cross-bridges. The three structures are remarkably similar. Backbone atom RMSDs over the β-sheet core are 0.7 and 0.9 Å for LytM and LytU, respectively, when overlaid with the lysostaphin CAT domain. Besides structural correspondence they share a similar dynamic behavior with a stable core and mobile catalytic site and surrounding loops (Raulinaitis et al., 2017a,b).

The CWT domain is compact, stabilized by extensive hydrogen bonding within loops as well as between loops and the rest of the molecule, and high similarity is found between the solution and crystal structures. Backbone and heavy atom RMSDs are 0.5 and 1.1 Å, respectively, for all residues in the closest matching pair. Some dynamic features are, however, observed. Micro- to millisecond time scale motions are present in five residues, K412, T428, R476, G486, and L488 and four residues, G401, K406, N466, S467 are prone to fast exchange with solvent, all in loops (Figure 1B).

### Lysostaphin domains do not interact *in vitro*

No inter-domain NOE peaks were observed in the NOESY spectra, strongly indicating that the two domains do not possess a stable interaction interface. The lack of chemical shift differences between the isolated CWT domain and the CWT domain in full-length lysostaphin (Supplementary Figure 2) provides additional solid evidence for non-interacting domains. Nevertheless, we further inspected the possibility of domain interaction by replacing the CAT-bound zinc cation with manganese and analyzing peak bleaching in a ^1^H, ^15^N HSQC spectrum. Unpaired electrons in isotropic paramagnetic probes such as manganese induce transverse paramagnetic relaxation enhancement (PRE) leading to line-broadening, affecting peak intensity, up to 35 Å from the paramagnetic center depending on the paramagnetic probe (Clore and Iwahara, 2009). In Mn^2+^-bound lysostaphin peaks disappeared or broadened only in the CAT domain (Supplementary Figure 3), at a maximum average distance of 23.6 Å from the paramagnetic center, leaving peaks from the far-most backside of this domain intact. Conceivably, as the linker allows such an arrangement, the domains could interact through this backside CAT domain region without the CWT domain peaks experiencing line-broadening.

The PRE experiment remaining inconclusive, we turned to the ^15^N relaxation data and overall rotational correlation times (τ_*c*_), which can also be used to untangle the confines of inter-domain mobility. The τ_*c*_s derived from ^15^N R_2_, R_1_ rates are notably different for the CAT and CWT domains, 7.8 and 6.0 ns at 35°C, respectively (Table 2). Also, the τ_*c*_ of the CWT domain in full-length lysostaphin is significantly higher than that of the isolated CWT domain, 4.1 ns. These indicate that there is a clear motional hindrance by the neighboring domain in full-length lysostaphin. There still is, however, substantial flexibility, considering that a unified complex encompassing both domains has a predicted τ_*c*_ of 13.4 ns.

**Figure 3:**
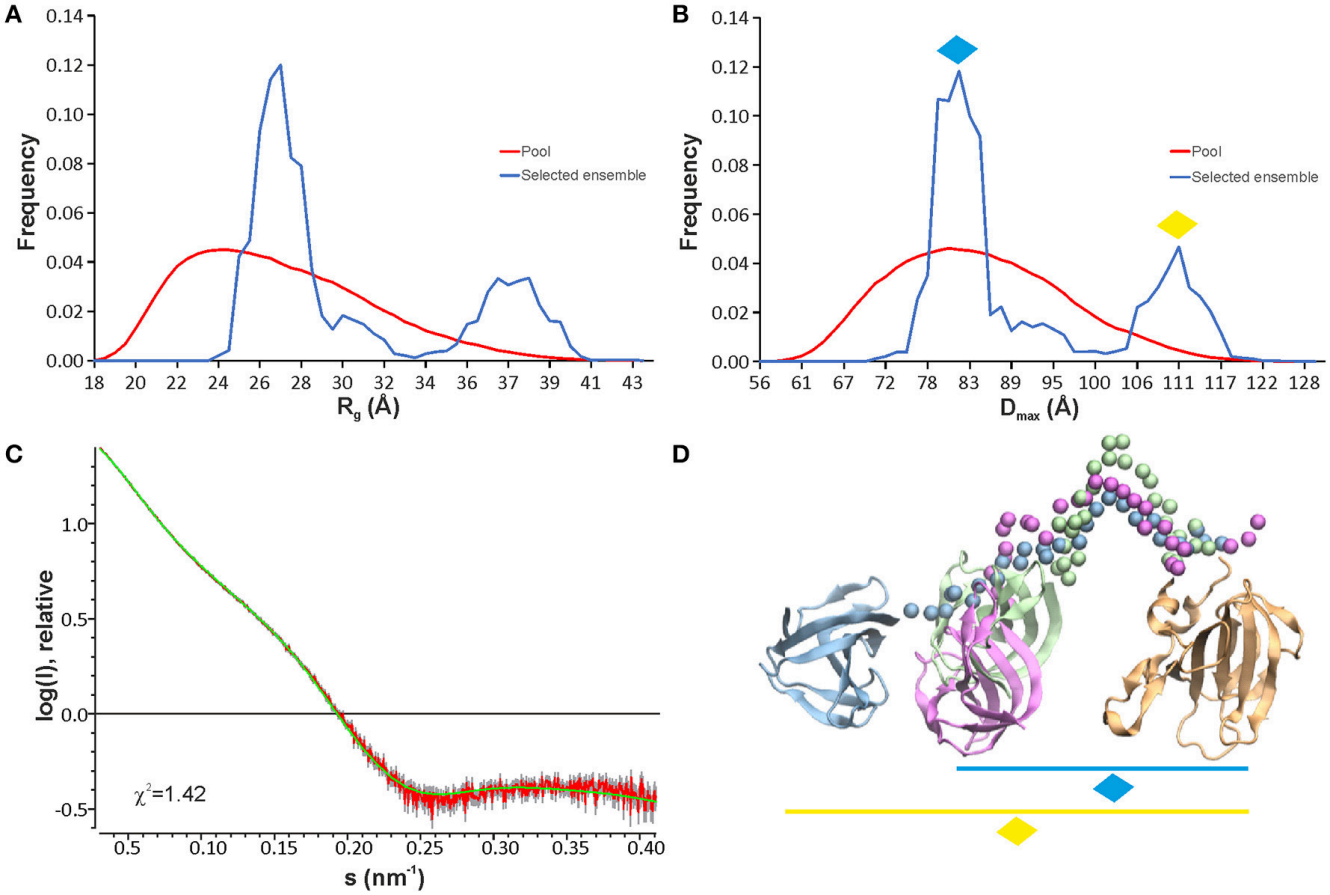
EOM analyses of lysostaphin SAXS data show that two conformation ensembles are present in solution as seen from R_g_ **(A)** and D_max_ **(B)** distributions. The pool represents the structures calculated based on the sequence and NMR structures of lysostaphin CAT and CWT domains. The selected models represent those structures within the pool that fit to the experimental scattering curve. **(C)** A typical fit obtained from the selected ensemble of structures to the experimental scattering of lysostaphin. **(D)** Three representative models of lysostaphin obtained from EOM calculations. The models are superimposed by the CAT domain (tan). CWT domains of three independent models are shown in green, magenta and blue. Green and magenta CWT domains belong to the compact conformations (marked with a blue diamond in **B**,**D**) and blue CWT domain to the extended conformations (marked with a yellow diamond in **B**,**D**). The CAT and the CWT domains of the NMR structure were used as rigid bodies in EOM calculations. Spheres represent amino acids in a random coil linker.

To further investigate the structure and flexibility of lysostaphin in solution, SAXS data were collected. An ensemble optimization (EOM) method was employed to analyse the SAXS data. In EOM calculations, lysostaphin CAT (residues 251-384) and CWT (410-493) domains of the NMR structure reported here were used as rigid bodies, and residues 385-409 as random coil. EOM runs yielded reproducible ensembles neatly fitting the experimental data with χ^2^-values around 1.4 (Figure 3C). The results clearly show that lysostaphin adopts two different conformations in solution; a more favored compact subpopulation with average *R*_*g*_ and *D*_*max*_ of 27 and 82 Å, respectively, and a less favored extended subpopulation, with average *R*_*g*_ and *D*_*max*_ of 37 Å and 110 Å (Figures 3A,B,D). The *D*_*max*_ of the predominant conformation is consistent with an average domain separation of 22–23 Å. This is considerably shorter than the length encompassed by a fully extended conformation of the linker containing two prolines, ~42 Å. This raises the possibility of the presence of secondary structure in the linker or interactions of the linker with one or other domain. Neither is, however, supported by secondary chemical shifts or NOE peaks. Moreover, significantly lower R_1_R_2_ products and heteronuclear NOEs found for linker residues K389-W402 with respect to the values in the CAT and CWT domains indicate that the linker is highly flexible. However, two sets of peaks were assigned for linker residues V394-P398, indicating the presence of two preferred conformations within this stretch, likely due to P398 cis/trans isomerism. This potentially offers a functional benefit to lysostaphin as it allows sampling of a larger conformational space and readjustment of domain orientations upon scission of pentaglycine bridges near its anchoring site (Aitio et al., 2012).

A dynamic inter-domain interaction in lysostaphin has been presented previously (Lu et al., 2013). Although the long flexible linker indeed allows the spatial proximity of the domains, there are no indications of such interaction in our data. The linker easily allows cleavage of adjacent and nearby pentaglycine bridges in PG near the bridge that serves as an anchor. Considering the architecture and dimensions of the PG lattice (Kim et al., 2015), formation of a CAT and CWT “envelope” around the very same target pentapeptide, which is also fixated between perpendicular peptidoglycan stem peptides that it connects, is very improbable due to steric restrictions. The current data remain in support of the flexible arrangements between partly independent domains.

### Binding of a second zinc by the catalytic histidines reduces lysostaphin catalytic activity

Proton NMR allows for direct and real-time reaction monitoring devoid of intricacies of cellular suspension (Raulinaitis et al., 2017a). Scission of pentaglycine by lysostaphin was monitored for 48 h by ^1^H NMR and under conditions of this study its catalytic rate appeared to be limited only by saturation with the substrate (Supplementary Figure 4). The substrate turnover rate by the enzyme in the first 3 h of the reaction is estimated to be in the order of 0.006 s^−1^.

**Figure 4:**
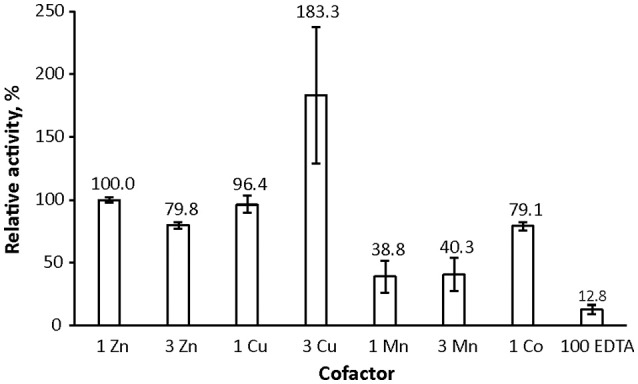
Lysostaphin activity dependence on zinc, cobalt, copper and manganese cofactors. Lysostaphin was incubated with pentaglycine (2.25 μM and 1 mM concentrations, respectively) in PBS buffer, pH 7.2, for 21 h at 37°C. Total cofactor ratios to protein were 1:1 and 3:1, EDTA excess was 70-fold. Purified enzyme, despite extensive treatment with EDTA during purification to remove any cofactors bound to the enzyme, nevertheless displayed residual activity. Activity was adjusted to one zinc-bound lysostaphin as 100% reference point. Bars represent standard error of the mean (S.E.M) of at least three independent measurements.

We used the method to assess lysostaphin catalytic activity dependence on different metal cofactors (Figure 4). Samples were first incubated for 21 h and then quenched by heating, followed by acquisition of a ^1^H spectrum. Results indicate that metal cations Zn^2+^, Co^2+^, and Cu^2+^ are suitable cofactors of lysostaphin, whereas Mn^2+^ is a weak cofactor. The activity increases when Cu^2+^ is present in excess. This behavior might be explained by Cu^2+^ being a better cofactor with a lower affinity, the enzyme reaching full metal cation saturation at higher cation concentrations. M23 family endopeptidases are recognized as zinc-dependent enzymes, yet other metal ions have been found to partially restore their activity (Firczuk et al., 2005; Wang et al., 2011) and recently Co^2+^ ions have been shown to yield a hyperactive LytU (Raulinaitis et al., 2017a). A partial inhibition of lysostaphin activity is observed in the excess of zinc. Even under the inhibition by an extra zinc ion, the enzyme retains its fold, as demonstrated by its ^1^H, ^15^N HSQC spectrum displaying features of a structured protein, very similar to those of the one-zinc form (Supplementary Figure 5). Notably, chemical shift perturbations, CSPs, are observed for peaks of residues in the catalytic groove as well as the surrounding loops. Changes in the dynamics are also observed (Figure 2). The majority of the largest changes occur for residues having the largest exchange contribution in the one-zinc form, located in the vicinity of the catalytic site. At these sites, the R_1_R_2_ products decrease, which indicates reduction of exchange in the μs-ms timescale, and structure stabilization. Loop amides remain unprotected from fast solvent exchange, though, as no additional peaks appear into the ^1^H, ^15^N HSQC spectrum. Conclusively, two additional cross-peak sets appear into a ^1^H, ^15^N HMBC spectrum with ^15^N chemical shifts corresponding to those of a zinc-coordinating nitrogen (Banci et al., 1998). Hence, binding of a second zinc, coordinated by the catalytic histidines, reduces catalytic activity.

The inhibitory effect of second zinc binding was also demonstrated and explained by the arrest of catalytic histidines in the autolytic LytU, where the process may have a regulatory role (Raulinaitis et al., 2017a). Whereas it is conceivable for an intracellular autolysin, such regulation by inhibition would be irrational for an extracellular bactericide like lysostaphin and is likely physiologically irrelevant.

### Pentaglycine interacts transiently with the N-terminal groove in the CWT domain

**Figure 5:**
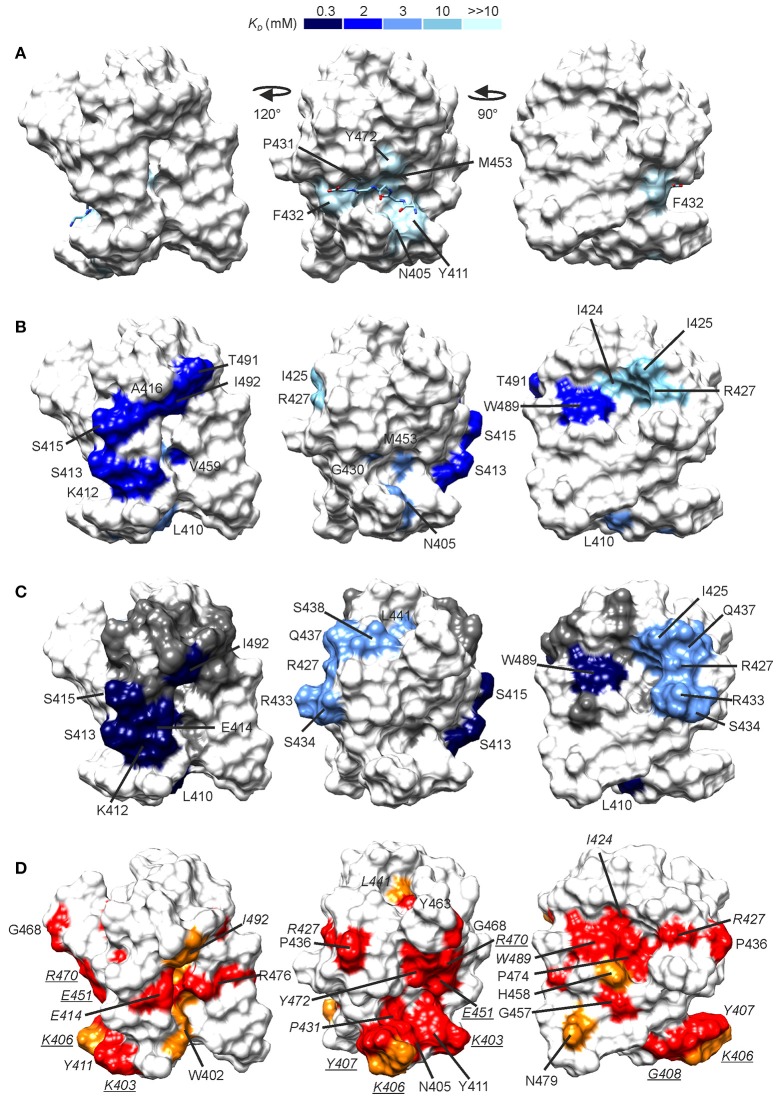
Peptide binding sites as revealed by NMR titrations. CSPs induced by G_5_K **(A)**, A-d-EK-GGGGG-A-d-EK-d-A **(B)**, and A-d-γ-EK-d-A-d-A **(C)** mapped onto the CWT domain structure. The surfaces are colored by the associated dissociation constant, the scale of which is given on the top of the figure. In **(A)**, the location of the bound pentaglycine is given from a structural superimposition of the free NMR structure and the X-ray CWT–substrate complex with PDB ID 5LEO. In **(C)**, between the high- and low affinity sites lies a group of residues which demonstrated a curved peak movement (see Supplementary Figure 4).These are shown in gray. **(D)** Conserved residues in *S. aureus*-targeting CWT domains mapped onto the lysostaphin CWT domain structure. Conservation was determined from a sequence alignment with ClustalW (Larkin et al., 2007) of lysostaphin CWT-like *S. aureus*-targeting sequences in the UniProt database (The Uniprot Consortium, 2017). In red are shown fully conserved residues and in orange residues with strongly similar properties. Conserved residues for which large CSPs were observed are labeled in italics, whereas conserved residues for which smaller CSPs were observed (0.06–0.09 ppm, not colored in A-C for clarity) are labeled in underlined italics. Sequence alignment is given in Supplementary Figure 5.

NMR suits particularly well to study weak or transient molecular interactions. We performed CWT domain binding site mapping with hexapeptides G_5_K and KG_5_ (Figure 5A and Supplementary Figure 6). The presence of lysine significantly enhanced the solubility of the peptide, which was essential in order to reach the large excess of ligand needed to achieve noticeable CSPs. The two pentaglycine derivatives produced similar amide peak CSPs when present in ~170 molar excess. Ligand interaction with CWT was independent of the presence of the CAT domain, as deduced by the similar CSPs observed for the CWT domain in full-length lysostaphin in the presence of ~140 molar excess of KG_5_ (data not shown).

Largest peak shifts, Δδ ≥ 0.10 ppm, in the presence of G_5_K were observed for N405, Y411, G430-F432, V452, M453, and Y472. Residues in strands β1 and β2 and the loop in between (K403-K412) form the bottom of the pentaglycine binding groove, and residues G430-F432 from the loop between strands β3 and β4 and Y472 in strand β7 the ceiling of the groove. Binding site mapping demonstrates CSPs for a delimited set of residues, without secondary, rearrangement-induced perturbations. Our data are nicely in accordance with the crystal structure of the lysostaphin CWT–pentaglycine complex (PDB ID 5LEO), which reveals that only very small structural rearrangements, namely few sidechain reorientations, occur upon binding of pentaglycine. The dissociation constant of the interaction is estimated to be ultra-weak, > 10 mM, in line with those observed for other pentapeptide–SH3b-type CWT domain interactions (Gu et al., 2014; Benešík et al., 2017).

The ^15^N relaxation data show interesting differences between free and pentaglycine-bound three-zinc lysostaphin. Both domains in the complex have larger τ_*c*_s than those in the free three-zinc form, 8.6 vs. 7.9 ns for the CAT and 6.3 vs. 5.9 ns for the CWT domains (Table 2). The difference is significant and cannot be attributed to the change in molecular weight only, considering that an increase of ~430 Da in molecular weight is predicted to increase τ_*c*_ by 0.2 ns only. Exchange between the complex and free forms could be the source of the larger τ_*c*_s, and indeed many local changes in the R_1_R_2_ products are observed. However, there is an equal number of positive and negative changes with values larger than < R_1_R_2_> ±1 SD, and the average R_1_R_2_ products remain the same for both domains. We gather that the additional rise can be interpreted in terms of transient inter-domain interaction in the presence of the ligand, or as a change in the degree of anisotropy of molecular tumbling induced by peptide or inter-domain interaction. Future studies in alignment media will help to derive information about relative domain orientation and inter-domain motions. The linker region shows similar dynamics in the absence and presence of the peptide, revealing that this stretch remains flexible, loosely connecting the domains.

### The CWT domain shows higher affinity toward the stem peptide

We reasoned that *in vivo* the CWT–pentaglycine interaction is likely to be bolstered by auxiliary contacts from the glycan backbone or the stem peptides in the PG lattice (for a description of the chemical structure of PG see Vollmer et al., 2008). We found that the CWT domain had no significant affinity for the muramyl dipeptide *N*-acetylmuramyl-l-A-d-γ-Q hydrate as deduced by the very small CSPs observed at ~80 molar excess of the peptide (data not shown).

Titration of the CWT domain with a synthetic peptide A-d-EKGGGGGA-d-EK-d-A, which would resemble the stem-cross-bridge-stem sequence in native PG, resulted, instead, in significant CSPs at considerably lower concentration ratios, ~24 molar excess. In addition to CSPs of residues in the pentaglycine groove, CSPs were observed for two groups of peaks with distinct binding affinities. If viewing the CWT domain the pentaglycine groove laying horizontally toward the viewer, the N-terminus of the bound pentaglycine on the right (PDB ID 5LEO), then K412-S413, S415-A416, V459, T491, and I492 form an elongated patch on the right side of the domain (Figure 5B). K412 is located between the pentaglycine and the elongated sites. These residues have an average *K*_*D*_ of ~2.3 mM. Residues I424-I425 and R427 form a small patch on the left side of the CWT domain with an average *K*_*D*_ of ~10.5 mM. W489 is located between the elongated and the small sites, but with a *K*_*D*_ of ~2.3 mM belongs to the elongated site. The peptide is not large enough to reach simultaneously the three surfaces. Considering that residues in the pentaglycine site have a much higher affinity toward this peptide than to pentaglycine or KG_5_/G_5_K (average *K*_*D*_ of ~3.2 mM) we suspect that one peptide molecule binds via its tail to the elongated site, and boosts the affinity of the pentaglycine site for the pentaglycine moiety of the peptide.

**Figure 6:**
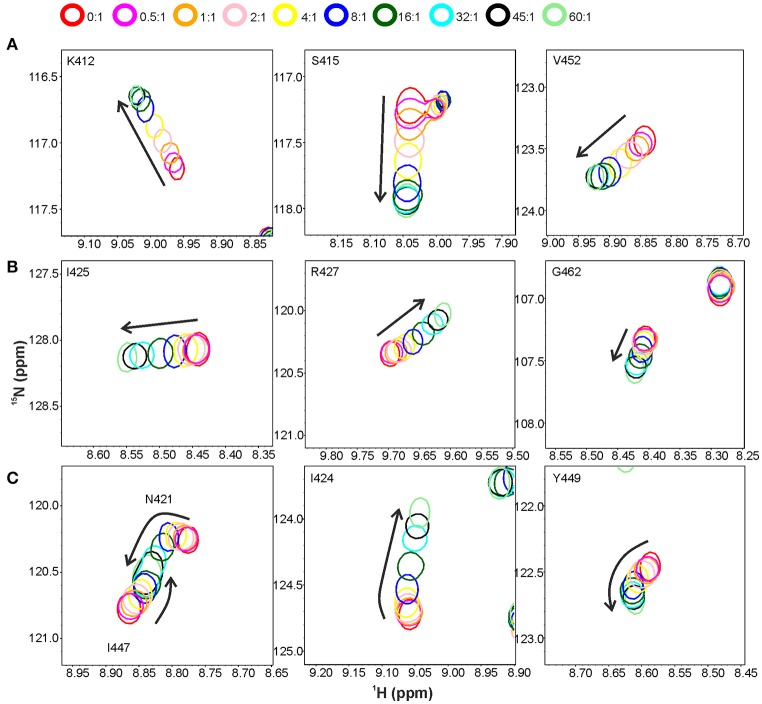
Titration of the CWT domain with the peptide A-D-γ-EK-D-A-D-A. Shown are overlaid sections of ^1^H, ^15^N HSQC spectra recorded at peptide to protein concentration ratios of 0:1 (red contours), 0.5:1 (magenta), 1:1 (orange), 2:1 (pink), 4:1 (yellow), 8:1 (blue), 16:1 (dark green), 32:1 (cyan), 45:1 (black) and 60:1 (light green). Three types of peak movement were observed: **(A)** linear, with saturation at ~32:1 concentration ratio, **(B)** linear, with saturation at ~60:1 concentration ratio, and **(C)** non-linear peak movement.

Titration with a shorter peptide A-d-γ-EK-d-A-d-A resulted in a complex peak behavior with three distinct patterns of peak position movement (Figure 6). Cross-peaks of residues L410, K412-S415, V452, W489, and I492 moved linearly upon peptide addition and reached saturation at ~32 molar excess of peptide. The dissociation constant was determined to be ~0.3 mM, notably lower than that for pentaglycine or the longer peptide. These residues mostly overlap with the elongated site of the previous titration (Figure 5C). Cross-peaks of the second group of residues also moved linearly, but began prominent movement and reached saturation at later steps of the titration, resulting in a dissociation constant of ~3 mM. These residues form a patch on the surface, which covers the small site of the previous titration (I425, R427, and L473) and has extensions of polar residues (R433, S434, Q437, and S438). The third group of peaks exhibited non-linear peak movement. For some of these residues the CSP are as large as for those in the two other groups, which rules out unspecific multiple binding as the cause of nonlinearity. Instead, as these residues are located between the first two surface patches, and include residues in the hydrophobic core, a conformational rearrangement brought about binding to the high-affinity site might be responsible for the curved titrations. Core residues V461 and V475 are probably involved in this rearrangement because their methyl peaks substantially shift in the ^1^H spectra along the titration (Supplementary Figure 7).

The importance of R296 and W358 in ALE-1 SH3b binding to *S. aureus* PG has been demonstrated by selective mutations (Lu et al., 2006). Binding of ALE-1 R296A and W358A mutants was reduced 3- and 2-fold, respectively, as compared to wild type binding. These residues correspond to R427 and W489 of lysostaphin CWT, belonging to the lower- and higher affinity sites, respectively, although structurally very close on the surface of CWT. R427 and W489 are strictly conserved in *S. aureus*-targeting SH3b domains (Figure 5D, Supplementary Figure 8). Based on the titration results and residue conservation, other residues which are likely to have an impact on CWT PG binding other than those in the pentaglycine binding groove are E414 and I492 of the high-affinity site.

We generated model structures of the CWT domain in complex with pentaglycine-stem peptide fragments (Supplementary Figure 9) with the assumption that the pentaglycine moiety occupies the N-terminal binding groove as in the structure 5LEO. The models indicate that the stem peptides on either side of pentaglycine seem to be too short to reach the CSPs observed in the presence of the A-d-γ-EK-d-A-d-A peptide. Also, CSPs in the region of the linkage between pentaglycine and the stem peptide are missing from the CWT domain surface. We gather that the observed CSPs might arise from a structural rearrangement occurring upon binding (see before), or be the result of stem peptide contacts present within the PG architecture, that is from stem peptides not directly linked to the pentaglycine recognized by the N-terminal groove. According to the parallel-stem architecture proposed by Kim et al. (2015) for *S. aureus* PG, contacts with stem peptides from further up or down in the densely packed glycan chains are probable.

These results clearly support the idea of formation of affinity-heaving accessory contacts with the PG network. Altogether it is advisable to consider using larger PG fragments in SH3b interaction studies in order to overcome the very low affinity of these domains toward pentaglycine (Gu et al., 2014; Benešík et al., 2017).

CWT–PG association studies have demonstrated that CWT binding to *S. aureus* cells cannot be blocked with excess of pentaglycine (Gründling and Schneewind, 2006), which is in accord with the extremely low affinity found for the pentaglycine–CWT interaction in the present study. The authors also demonstrated that inhibition of binding was achieved only with PG fragments containing multiple cross-linked murein subunits, not with smaller murein mono- or dimers. This suggests that the CWT domain affinity for *S. aureus* PG has to be higher than that observed here for the five-residue peptide, ~300 μM. It also suggests that the PG lattice structure is needed for maximal affinity, only cross-linked fragments with multiple murein moieties being capable of reproducing this structure. In other words, binding to the pre-arranged PG lattice is likely to be entropically more favorable than binding, through induced folding, to a disordered peptide nonetheless containing the same interaction sites.

## Conclusions

This study reveals details on structure and dynamics within and between the lysostaphin domains, on metal ion preference of the CAT domain, as well as characterizes substrate binding sites and affinities of the CWT domain. Remarkably, the affinity of the CWT domain was found to be much higher toward the stem peptide than toward pentaglycine. Also, the CWT domain affinity for pentaglycine increased when the latter was annexed to a stem peptide-like moiety. These findings underscore the importance of auxiliary contacts in CWT PG recognition. The stem peptide analogs interacted with two surfaces on both sides of the N-terminal pentaglycine binding groove of the CWT domain. Residues E414, R427, W489, and I492 are predicted to be crucial to the PG interaction. Taking into account the PG architecture, rather than just the pentaglycine bridge, is imperative to appreciate the factors contributing to the catalytic activity of the CAT domain, to recognize the structural components influencing the interaction between lysostaphin and PG and to engineer novel lysostaphin- or CWT-based antimicrobials.

## Data availability statement

The assigned chemical shifts and coordinates of lysostaphin have been deposited in the BioMagResBank (http://www.bmrb.wisc.edu/) and the PDB (https://www.rcsb.org/) with accession codes 34121 and 5NMY, respectively.

## Author contributions

PP conceived and designed the research. VR and LK produced and purified the proteins. VR and HM performed enzyme activity studies. UP performed SAXS measurements and SAXS data analysis. HT carried out MD simulations. HT, LK, and PP conducted CWT domain NMR studies. HT and PP performed full-length lysostaphin NMR studies. All authors wrote the paper.

### Conflict of interest statement

HM was employed by company VTT Technical Research Centre of Finland Ltd. The remaining authors declare that the research was conducted in the absence of any commercial or financial relationships that could be construed as a potential conflict of interest.
