# Supplementary material for: Structural and Functional Insights Into Lysostaphin–Substrate Interaction
Source: Front Mol Biosci. 2018 Jul 3;5:60. doi: 10.3389/fmolb.2018.00060 (PMC6038053; doi:10.3389/fmolb.2018.00060)
Supplement: Supplementary file 1 [file Presentation_1.PDF]

Supplementary Material

Structural and Functional Insights into Lysostaphin–Substrate Interaction

Helena Tossavainen, Vytas Raulinaitis, Linda Kauppinen, Ulla Pentikäinen, Hannu Maaheimo, Perttu Permi*

*** Correspondence:** Corresponding Author: perttu.permi@jyu.fi

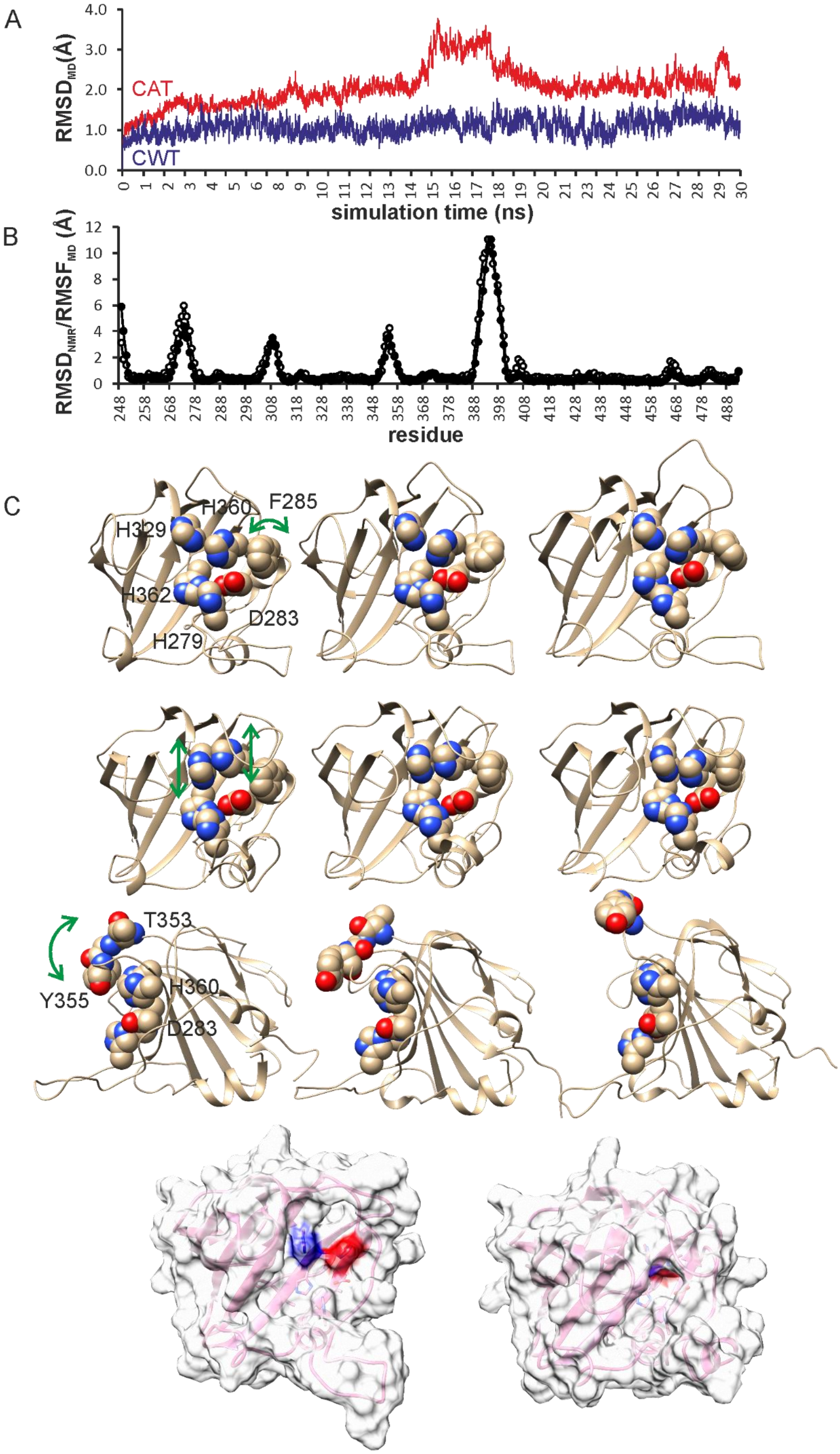
A
4.0
3.0
2.0
1.0
0.0
RMSD$_{MD}$(Å)
CAT
CWT
0 1 2 3 4 5 6 8 9 10 11 12 13 14 15 16 17 18 19 20 21 23 24 25 26 27 28 29 30
simulation time (ns)
B
12
10
8
6
4
2
0
RMSD$_{NMR}$/RMSF$_{MD}$ (Å)
248 258 268 278 288 298 308 318 328 338 348 358 368 378 388 398 408 418 428 438 448 458 468 478 488
residue
C
H360
F285
H329
H362
D283
H279
T353
Y355
H360
D283

Supplementary Figure 1. (A) Ca RMSDs of the CAT and CWT domains with respect to the equilibrated conformation as a function of time. The peak in the CAT RMSD in the middle of the simulation originates from the movement of the N-terminal loop (residues Y267-G281). **(B)** Combined graph of Cα RMSF (white circles) derived from lysostaphin MD simulation and RMSD (black circles) from the lysostaphin NMR ensemble as a function of the amino acid sequence. **(C)** Trajectory snapshots from the catalytic histidines' (H329, H360) structural neighborhood during the MD simulation, revealing possible mechanisms contributing to the disappearance of their HMBC peaks. F285, next to H360, is able to rotate (first row). H329 and H360 can slide relative to each other (first and second row). The loop closest to the catalytic histidines (residues G351-P359) can adopt a multitude of conformations, ranging from it having close contacts with the catalytic histidines to it being at a distance of several Ångströms from them (first and third row). The last row demonstrates that the solvent accessibilities of the catalytic histidine rings vary significantly during the simulation. Shown are snapshots representing surfaces of conformations with high (left) and low exposure. H329 is highlighted in blue and H360 in red.

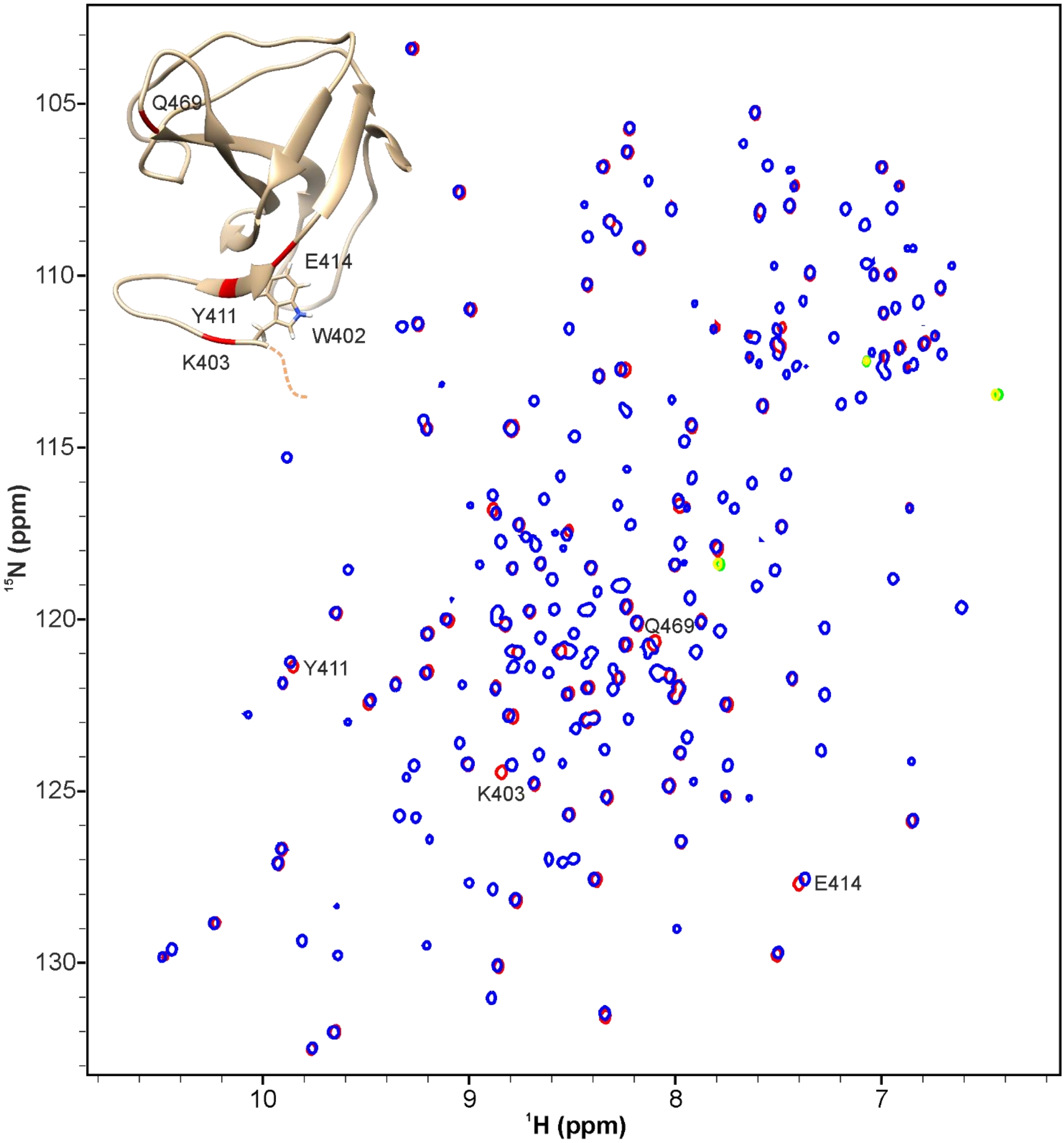

Supplementary Figure 2. Overlaid ^{1}H, ^{15}N HSQC spectra of single CWT domain (red contours, and green for aliased arginine ε groups) and CWT domain in full-length lysostaphin (blue/yellow contours). Labeled residues display Δδ ≥ 0.03 ppm. The largest peak shift is observed for K403, for which Δδ is 0.06 ppm. At top left these residues are highlighted in the CWT domain structure. W402 is the N-terminal residue in the CWT domain when expressed as a single domain. The origin of Q469 peak shift remains undetermined.

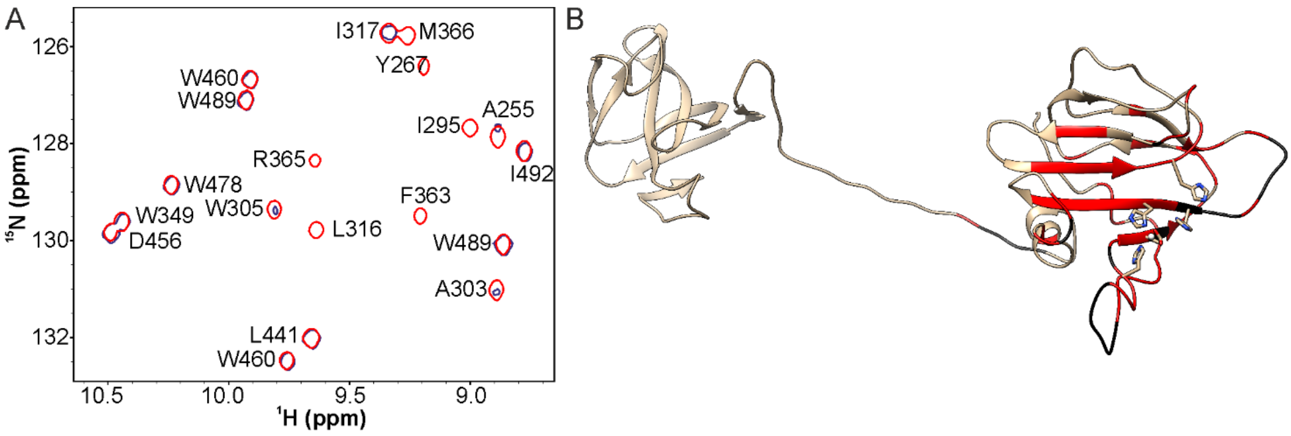

Supplementary Figure 3. Peak bleaching in Mn^{2+}-bound lysostaphin. **(A)** Overlay of ^{1}H, ^{15}N HSQC spectra of Zn^{2+}- (red contours) and Mn^{2+}-bound (blue contours) lysostaphin. A subset of the spectrum is presented, in which peak bleaching caused by the bound paramagnetic Mn^{2+} is clearly observed. **(B)** Amides experiencing peak bleaching mapped onto the protein structure. No bleaching was observed in the CWT domain (left). In the CAT domain (right), red color indicates amides whose peak have disappeared from the Mn^{2+} spectrum. In black are shown residues for which no data are available due to overlap or the presence of proline in the amino acid sequence. The cation is depicted as a grey sphere.

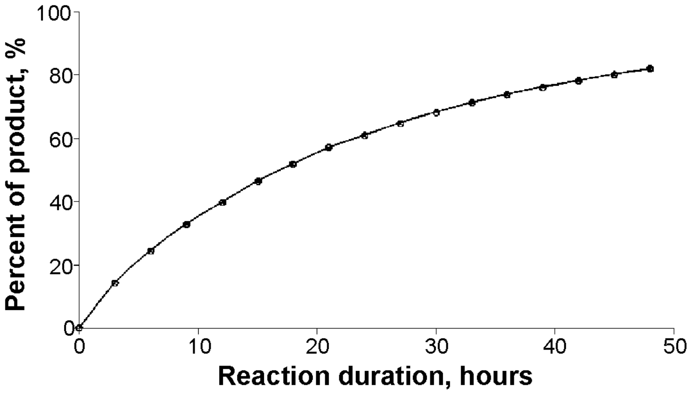

Supplementary Figure 4. Generation of pentaglycine cleavage product. Pentaglycine was incubated with lysostaphin (1 mM and 2.25 µM final concentrations, respectively) at 37 °C in PBS buffer, pH 7.2. Proton spectra were acquired every 3 hours for 48 h. Spectra were processed and peaks integrated using Bruker TopSpin 3.5 software. Bars represent standard error of the mean (S.E.M) of two independent measurements.

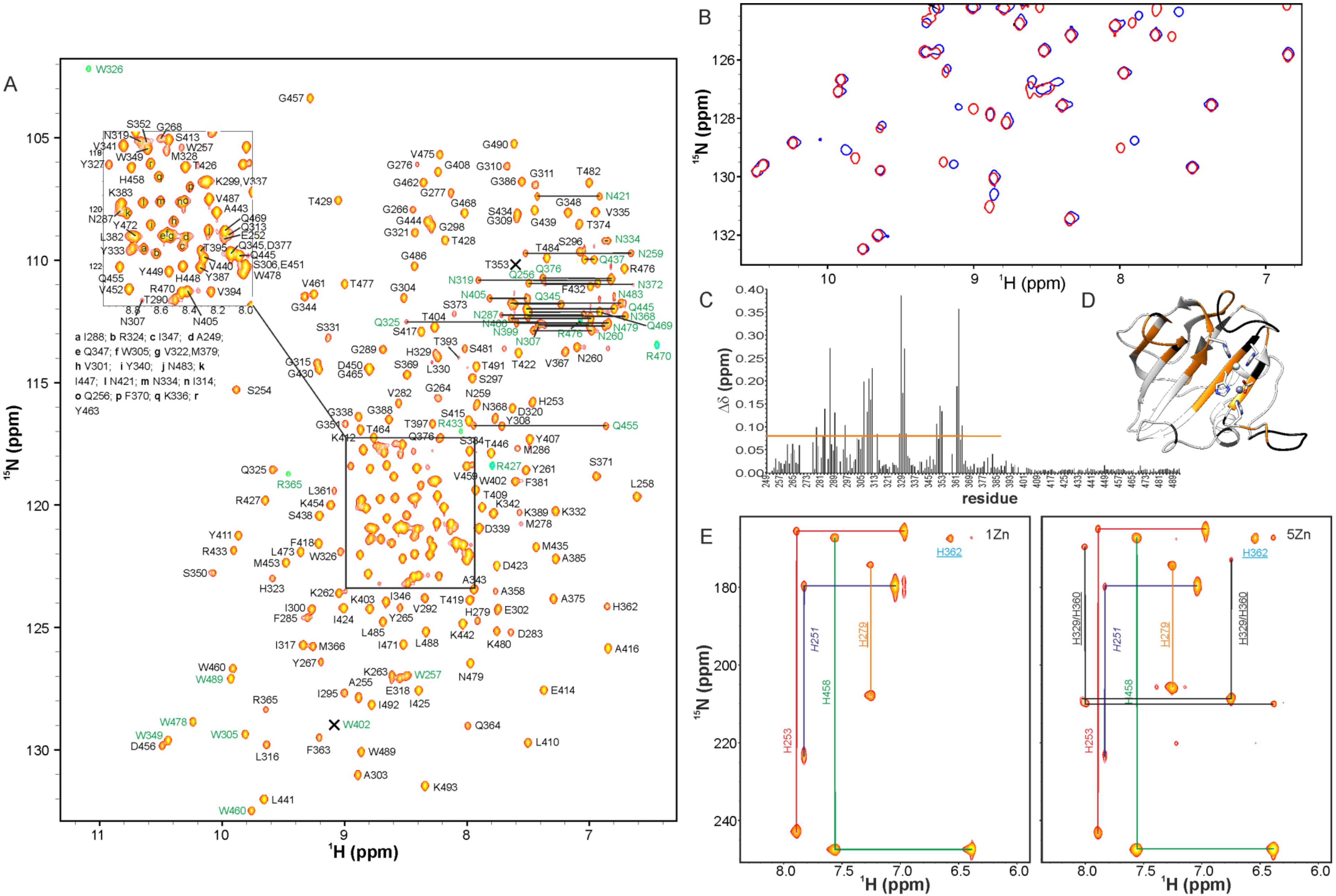
A
B
C
D
E
¹H (ppm)
¹⁵N (ppm)
Δδ (ppm)
residue
a I288; b R324; c I347; d A249;
e Q347; f W305; g V322,M379;
h V301; i Y340; j N483; k
I447; l N421; m N334; n I314;
o Q256; p F370; q K336; r
Y463
1Zn
5Zn
H362
H251
H279
H458
H253
H329/H360

Supplementary Figure 5. Comparison of spectra collected from samples with equimolar Zn^{2+} and lysostaphin concentrations and with excess of Zn^{2+}. **(A)** A 800 MHz ^{1}H, ^{15}N HSQC spectrum of one-zinc lysostaphin with resonance assignments annotated. Side chain signals have green labels. Asn and Gln side chain peaks are indicated by lines. W326 side chain peak is aliased and appears as a negative signal at the upper left corner of the spectrum. Arginines' ε groups also display aliased negative peaks. Peaks marked with crosses appear at lower contour levels. K293 amide displays a very weak peak at a peculiar ^{1}H shift near the water signal (4.95, 126.7 ppm). The inset shows an expansion of the crowded middle region of the spectrum. **(B)** A subset of overlaid ^{1}H, ^{15}N HSQC spectra of samples with 1:1 (red contours) and 3:1 (blue contours) Zn^{2+} to lysostaphin concentration ratios. **(C)** CSPs, calculated as $\Delta\delta=(\Delta\delta H^2+(0.154\times\Delta\delta N)^2)^{1/2}$, as a function of the amino acid sequence. **(D)** CSPs with $\Delta\delta>0.08$ are mapped onto the CAT domain structure. The presumed, approximate location of the second bound zinc cation is depicted as a white sphere. **(E)** Histidine ^{15}N region ^{1}H, ^{15}N HMBC spectra of samples with 1:1 (left) and 5:1 (right) Zn^{2+} to lysostaphin concentration ratios. From the peak pattern the tautomeric state can be determined for H253 (ε2-protonated) and H458 (δ1-protonated). The peak set connected with blue lines is tentatively assigned to an ε2-protonated H251. The Cδ2, Cε1 chemical shift difference gives the tautomeric state of the Zn^{2+}-coordinating H279 (δ1-protonated). H279 has overlapping Hδ2, Hε1 chemical shifts, and a Nε2 chemical shift typical for a zinc-coordinating histidine, ~210 ppm. For H362 only one signal at its Hδ2 shift is present in the one-zinc spectrum. When excess of Zn^{2+} is added two new peak sets appear (black lines), those of the catalytic histidines H329 and H360, both δ1-protonated. The tautomeric states of H323 and H448 have not been assigned.

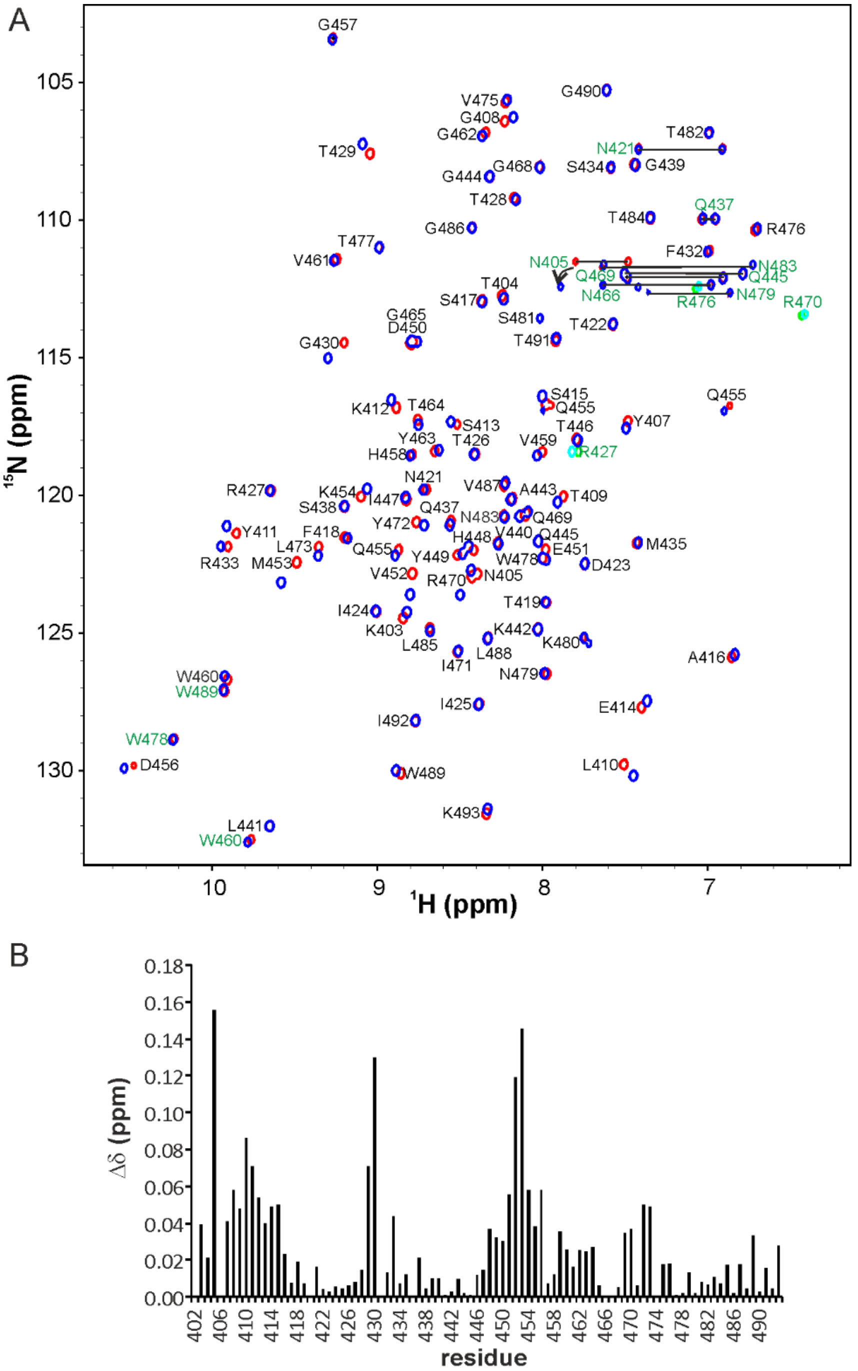

Supplementary Figure 6. (A) Overlay of spectra of the CWT domain acquired in the absence (red/green contours) and presence (blue/cyan contours) of ~170 molar excess the G_5K hexapeptide. Peaks originating from sidechains are labeled in green. Aliased arginine ε groups appear as negative peaks. Asn and Gln side chain peaks are indicated by lines. **(B)** Histogram of the observed CSPs, calculated as $\Delta\delta=(\Delta\delta H^2+(0.154\times\Delta\delta N)^2)^{1/2}$, as a function of the amino acid sequence.

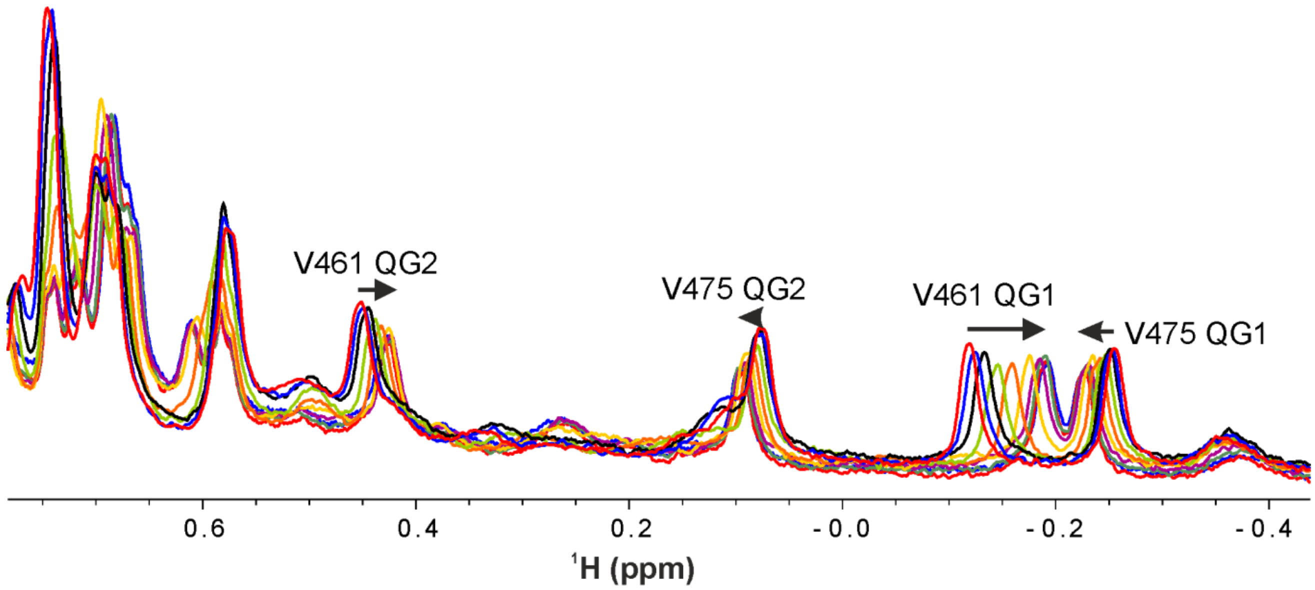

Supplementary Figure 7. Methyl peak movement of V461 and V475 in ^{1}H spectra acquired along the titration of the CWT domain with the peptide A-D-γ-EK-D-A-D-A.

```
LSSsim      402 WKTNKYGTLYKSESASFT-PNTDIITRTTGPFRSMPQSGVLKAGQTIHYDEVMKQDGHVW 460
LSSsta      389 WKTNKYGTLYKSESASFT-PNTDIITRTTGPFRSMPQSGVLKAGQTIHYDEVMKQDGHVW 447
ALE-1       271 YKTNKYGTLYKSESASFT-ANTDIITRLTGPFRSMPQSGVLRKGLTIKYDEVMKQDGHVW 329
LYTA        387 WKRNKYGTYYMEESARFTNGNQPITVRKVGPFLSCPVGYQFQPGGYCDYTEVMLQDGHVW 446
phi11       387 WKRNKYGTYYMEESARFTNGNQPITVRKVGPFLSCPVGYQFQPGGYCDYTEVMLQDGHVW 446
phiMR11     387 WKRNKYGTYYMEENARFTNGNQPITVRKIGPFLSCPVAYQFQPGGYCDYTEVMLQDGHVW 446
phiMR25     387 WKRNKYGTYYMEESARFTNGNQPITVRKIGPFLSCPVAYQFQPGGYCDYTEVMLQDGHVW 446
S13'        159 WKQNQYGTYYRNENATFTCGFLPIFARVGSPKLSEPNGYWFQPNGYTPYDEVCLSDGYVW 218
S24-1       156 WKQNQYGTYYRNENATFTCGFLPIFARVGSPKLSEPNGYWFQPNGYTTYDEVCLSDGYVW 215
44AHJD      156 WKRNQYGTYYRNENGTFTCGFLPIFARVGSPKLSEPNGYWFQPNGYTPYNEVCLSDGYVW 215
phageK      401 WKKNQYGTWYKPENATFVNGNQPIVTRIGSPFLNAPVGGNLPAGATIVYDEVCIQAGHIW 460
                :* *:*** *  *.. *.     * .*  .*  . * .  :  .    * **  . *::*

LSSsim      461 VGYTGNSGQRIYLPVRTWNKSTN---TLGVLWGTIK 493
LSSsta      448 VGYTGNSGQRIYLPVRTWNKSTN---TLGVLWGTIK 480
ALE-1       330 VGYNTNSGKRVYLPVRTWNESTG---ELGPLWGTIK 362
LYTA        447 VGYTWE-GQRYYLPIRTWNGSAPPNQILGDLWGEIS 481
phi11       447 VGYTWE-GQRYYLPIRTWNGSAPPNQILGDLWGEIS 481
phiMR11     447 VGYTWE-GQRYYLPIRTWNGSAPPNQILGDLWGEIS 481
phiMR25     447 VGYTWE-GQRYYLPIRTWNGSAPPNQILGDLWGEIS 481
S13'        219 IGYNWQ-GSRYYLPVRKWNGKTGNSYSVGIPWGVFS 253
S24-1       216 IGYNWQ-GTRYYLPVRQWNGKTGNSYSVGIPWGVFS 250
44AHJD      216 IGYNWQ-GTRYYLPVRQWNGKTGNSYSVGIPWGVFS 250
phageK      461 IGYNAYNGNRVYCPVRTCQGVPPN-QIPGVAWGVFK 495
                :**.   * * * *:*  :  .      *  ** :.
```

Supplementary Figure 8. Sequence alignment of SH3b domains. Sequences represent SH3b domains from *S. simulans* lysostaphin, *S. staphylolyticus* lysostaphin, *S. capitis* ALE-1, *S. aureus* autolysin LytA and *S. aureus*-targeting phages phi11, phiMR11, phiMR25, S13', S24-1, 44AHJD and phage K. Sequences were retrieved from the UniProt database using the BLAST tool with *S. simulans* lysostaphin SH3b domain sequence as the query sequence. Sequences were aligned using ClustalW. Fully conserved residues are marked with an asterisk and colored red whereas residues with strongly similar properties are marked with a colon and colored in orange. The colors correspond to those in Figure 5D. In bold are highlighted the residues which by selective mutations have been shown to be crucial to pentaglycine binding in ALE-1 CWT (Lu et al., 2006).

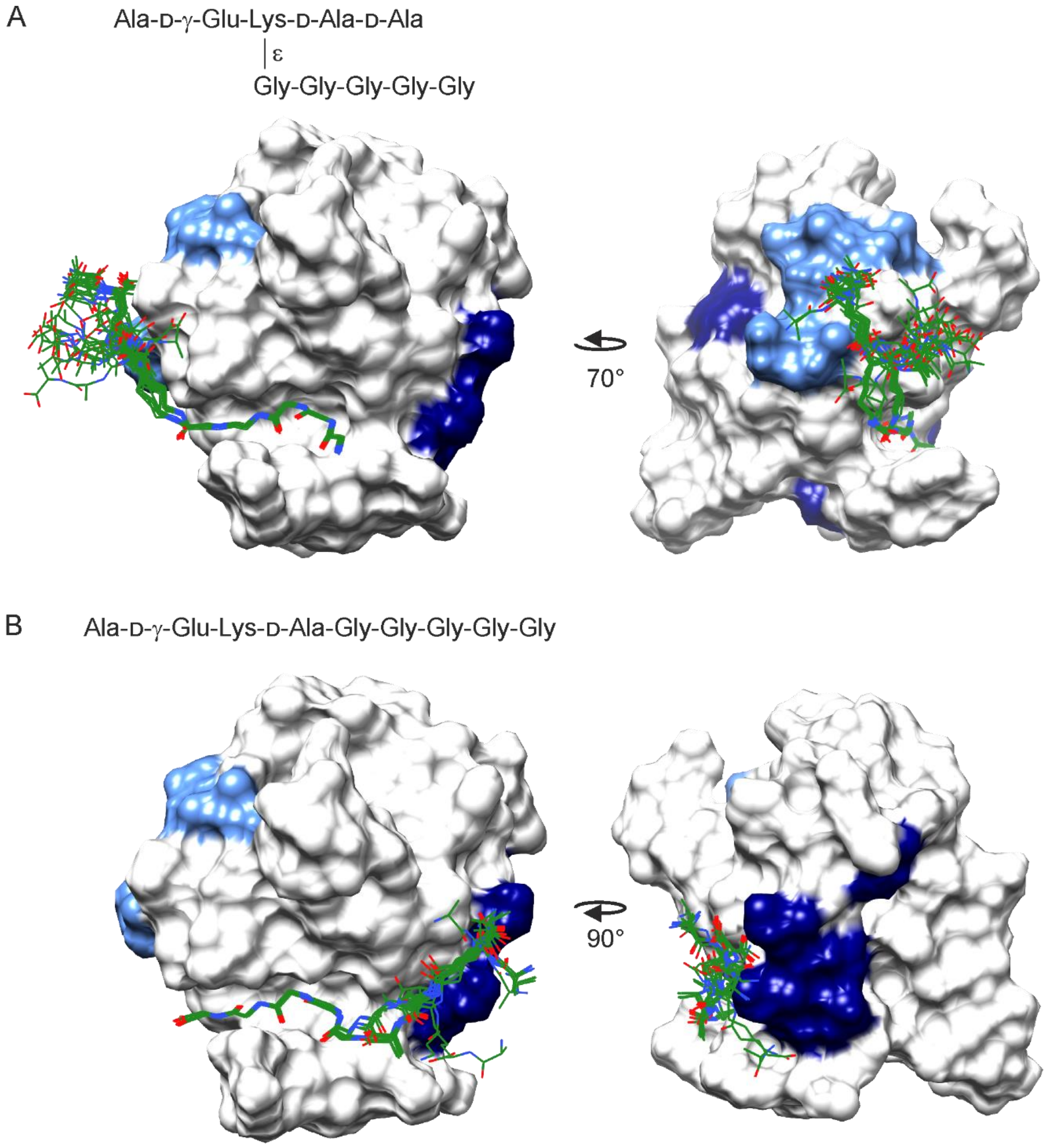

Supplementary Figure 9. Comparison of CSPs induced by the peptide A-D-γ-EK-D-A-D-A and model conformations of PG fragments encompassing pentaglycine and the stem peptide on the C-terminal side of pentaglycine, linked to the stem peptide lysine through the side chain ε group **(A)** or on the N-terminal side of pentaglycine **(B)**. The molecule on the right has been rotated by 70 degrees counterclockwise (A) or 90 degrees clockwise (B) relative to the molecule on the left. Models were calculated with CNS (Brunger et al., 1998; Brunger, 2007) keeping the location and dihedral angles of the pentaglycine moiety fixed to those observed in the 5LEO structure. Ambiguous NOE restrains, derived from the observed CSPs, were included between the stem peptide moieties and the CWT domain to guide the model complex structure generation. Twenty representative peptide conformations are presented. The color coding on the CWT domain surface is as in Figure 5, with dark blue representing the higher affinity site and light blue the lower affinity site in the A-D-γ-EK-D-A-D-A peptide titration.

References

Brunger, A.T., Adams, P.D., Clore, G.M., Gros, P., Grosse-Kunstleve, R.W., Jiang J.-S., et al. Crystallography & NMR System (CNS), A new software suite for macromolecular structure determination. (1998). *Acta Cryst. D Biol. Crystallogr.* 54, 905-921.

Brunger, A.T., Version 1.2 of the Crystallography and NMR System. (2007) *Nature Protoc.* 2, 2728-2733.

Lu, J.Z., Fujiwara, T., Komatsuzawa, H., Sugai, M., and Sakon, J. Cell wall-targeting domain of glycylglycine endopeptidase distinguishes among peptidoglycan cross-bridges. (2006). *J. Biol. Chem.* 281, 549-558. doi: 10.1074/jbc.M509691200
