# Supplementary figures and images for: Structural and Functional Insights Into Lysostaphin–Substrate Interaction
Source: Front Mol Biosci. 2018 Jul 3;5:60. doi: 10.3389/fmolb.2018.00060 (PMC6038053; doi:10.3389/fmolb.2018.00060)

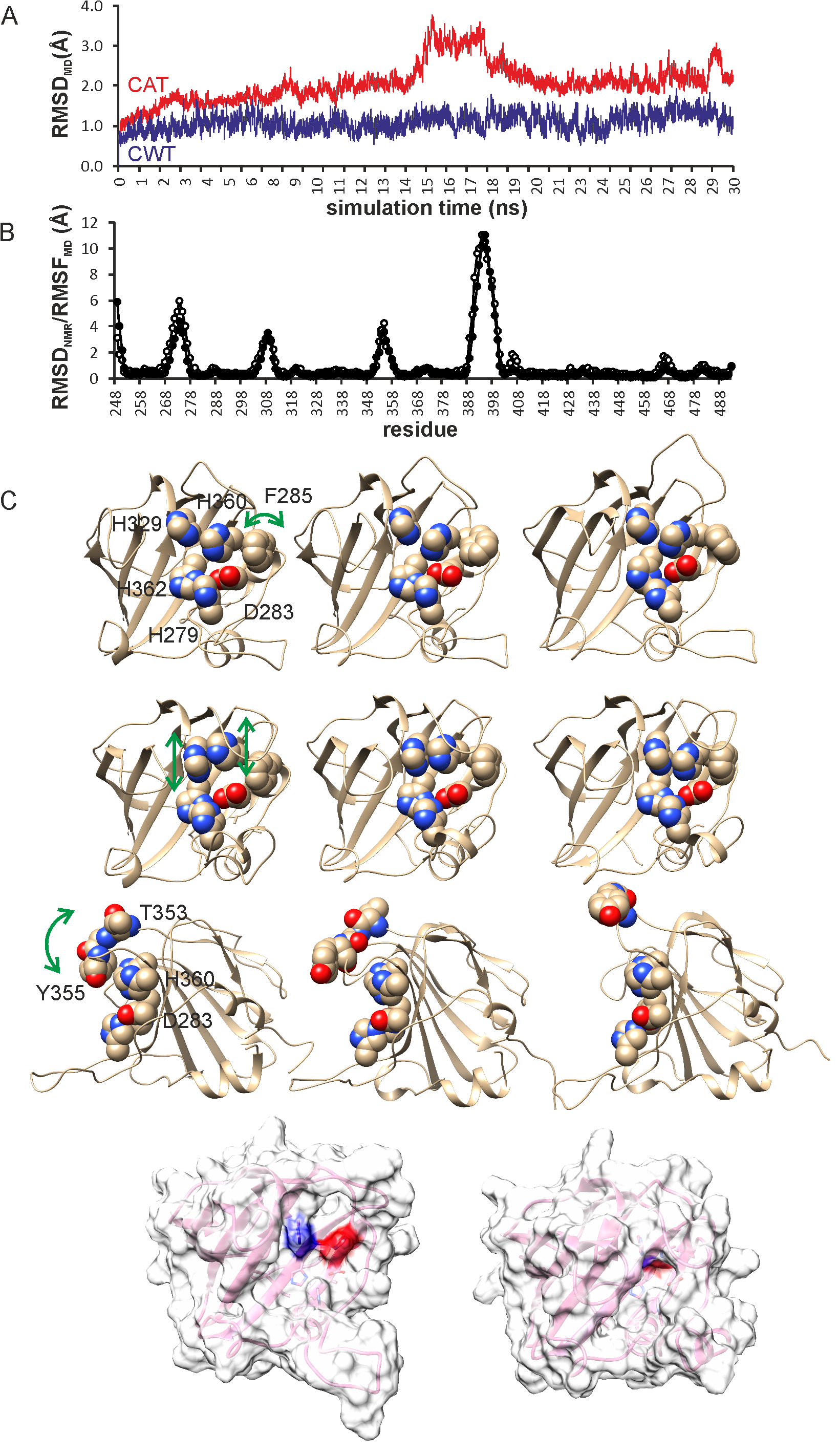

Supplement: Supplementary file 2 [file Image_1.TIF]

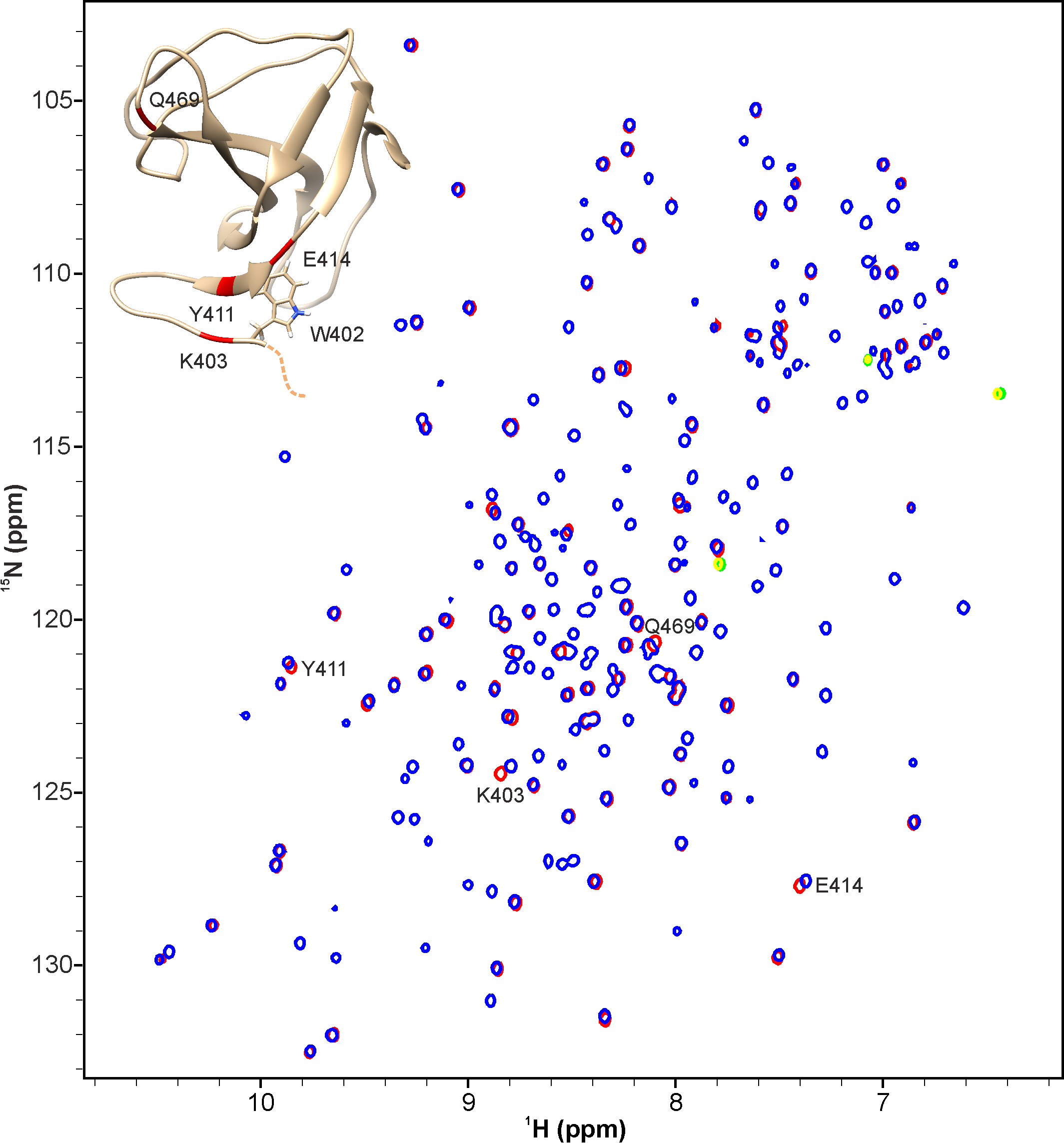

Supplement: Supplementary file 3 [file Image_2.TIF]

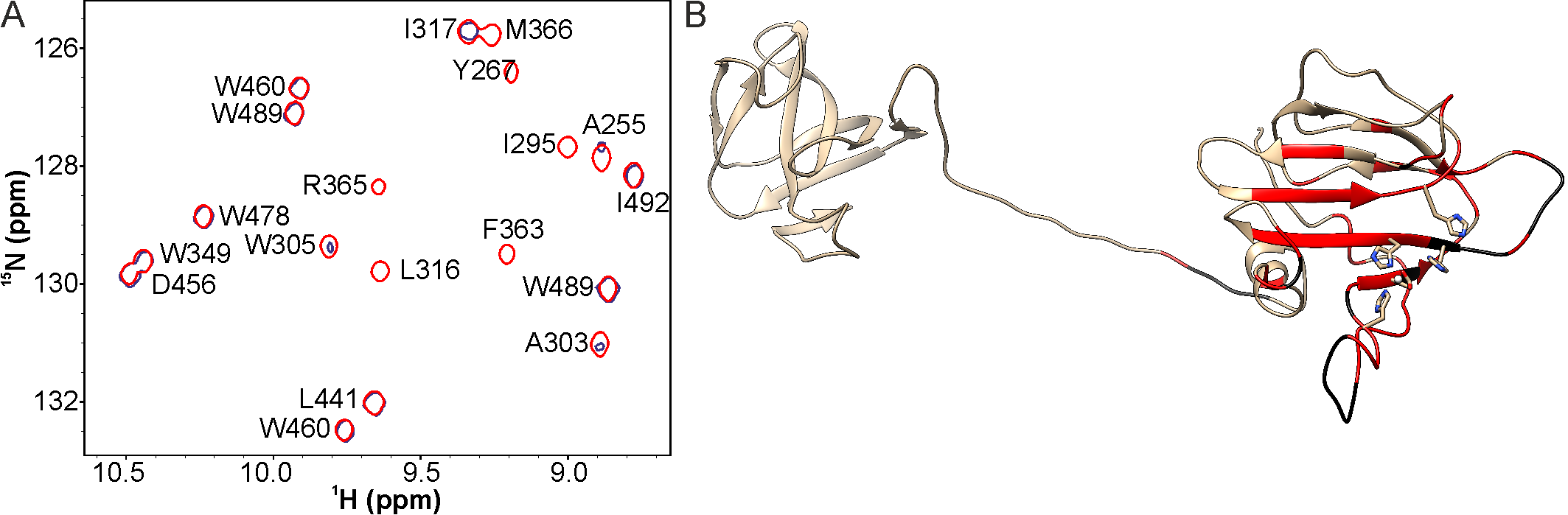

Supplement: Supplementary file 4 [file Image_3.TIF]

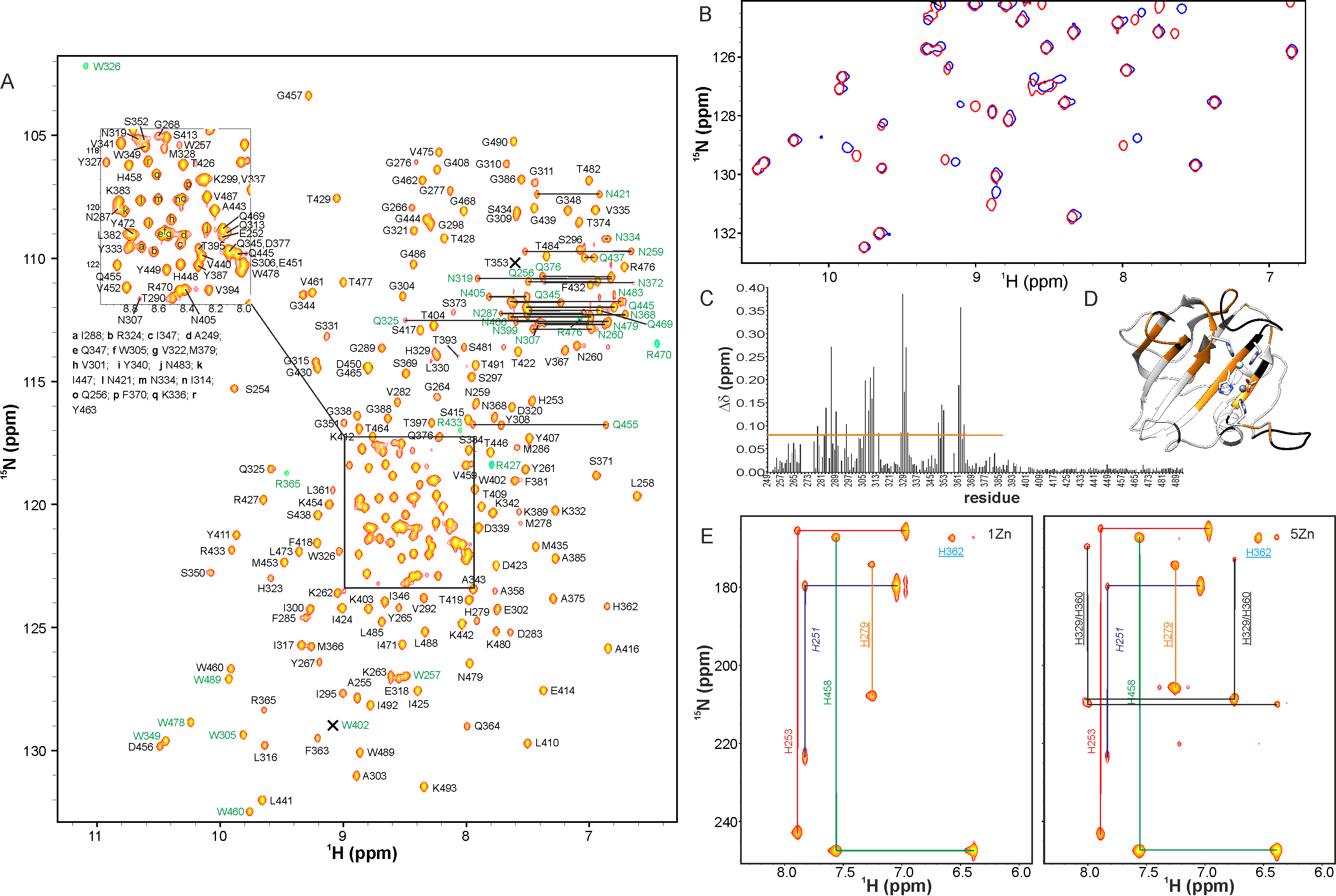

Supplement: Supplementary file 6 [file Image_5.TIF]

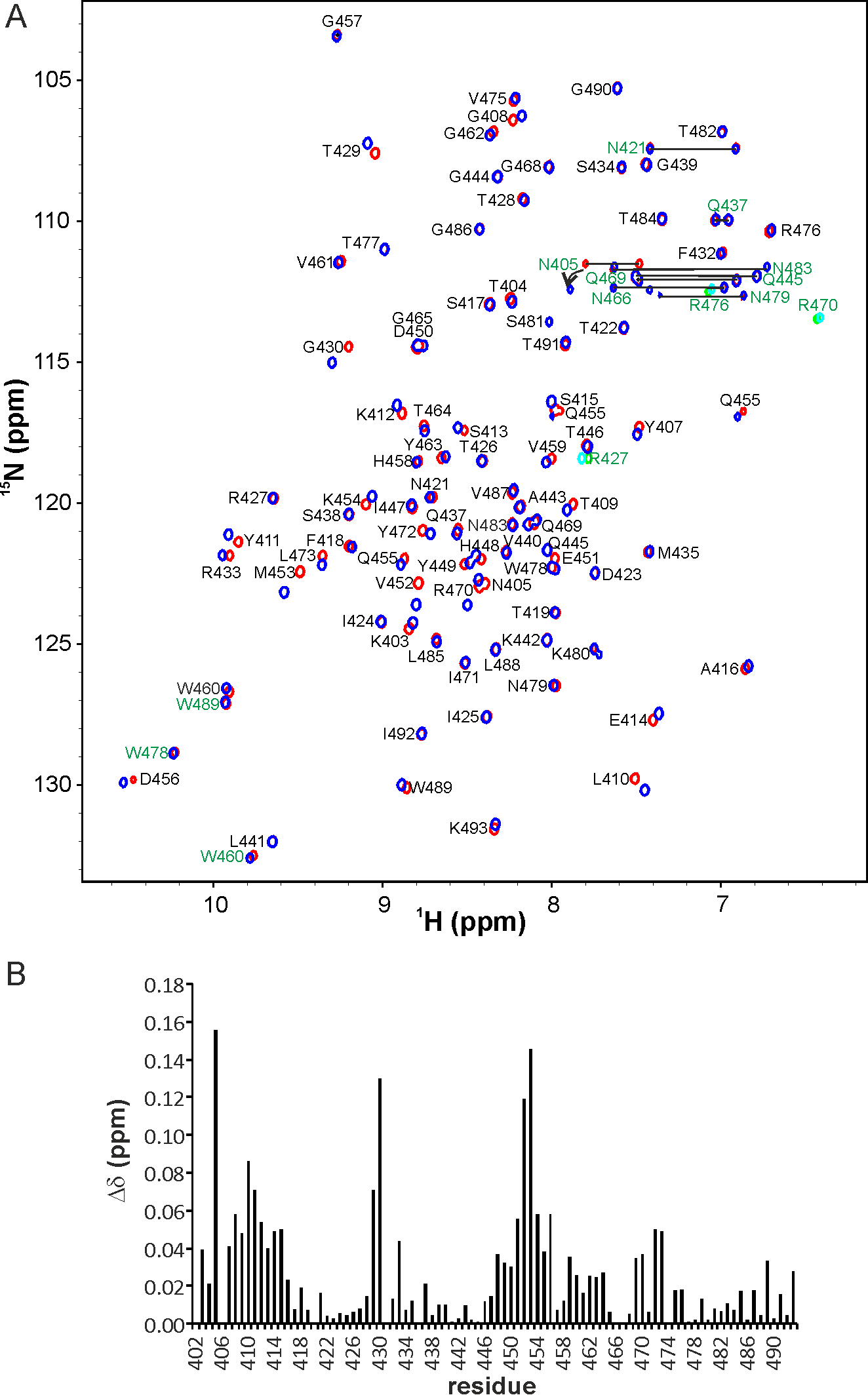

Supplement: Supplementary file 7 [file Image_6.TIF]

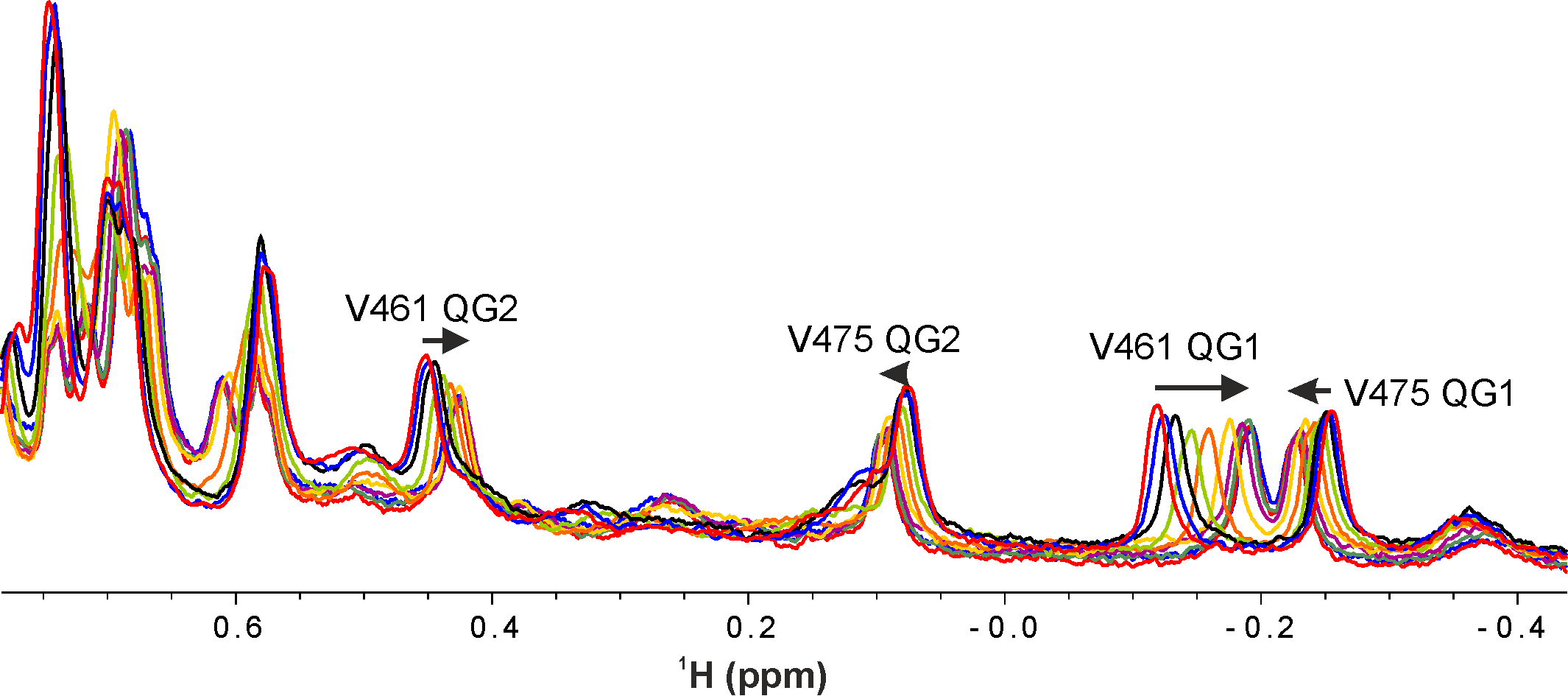

Supplement: Supplementary file 8 [file Image_7.TIF]

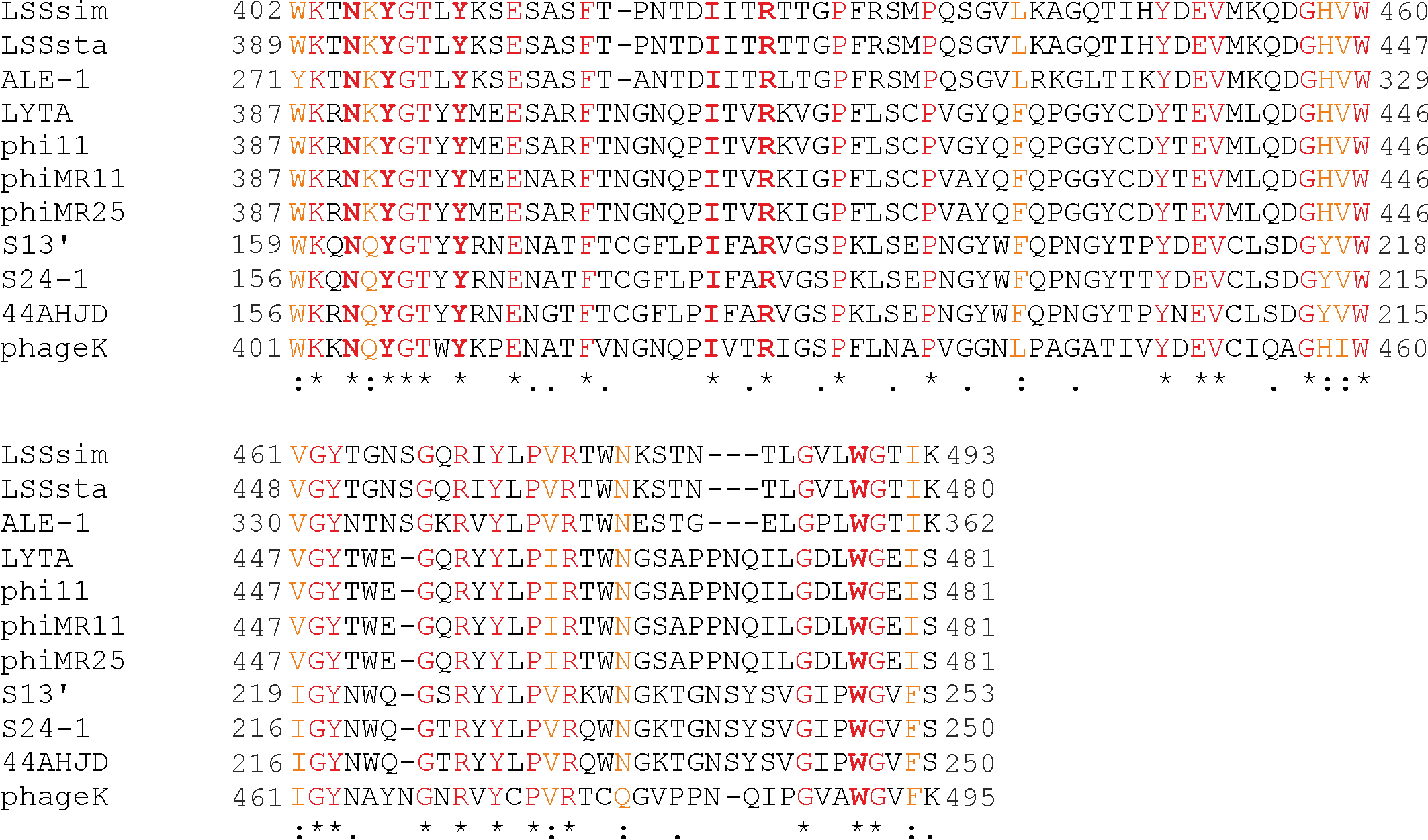

Supplement: Supplementary file 9 [file Image_8.TIF]

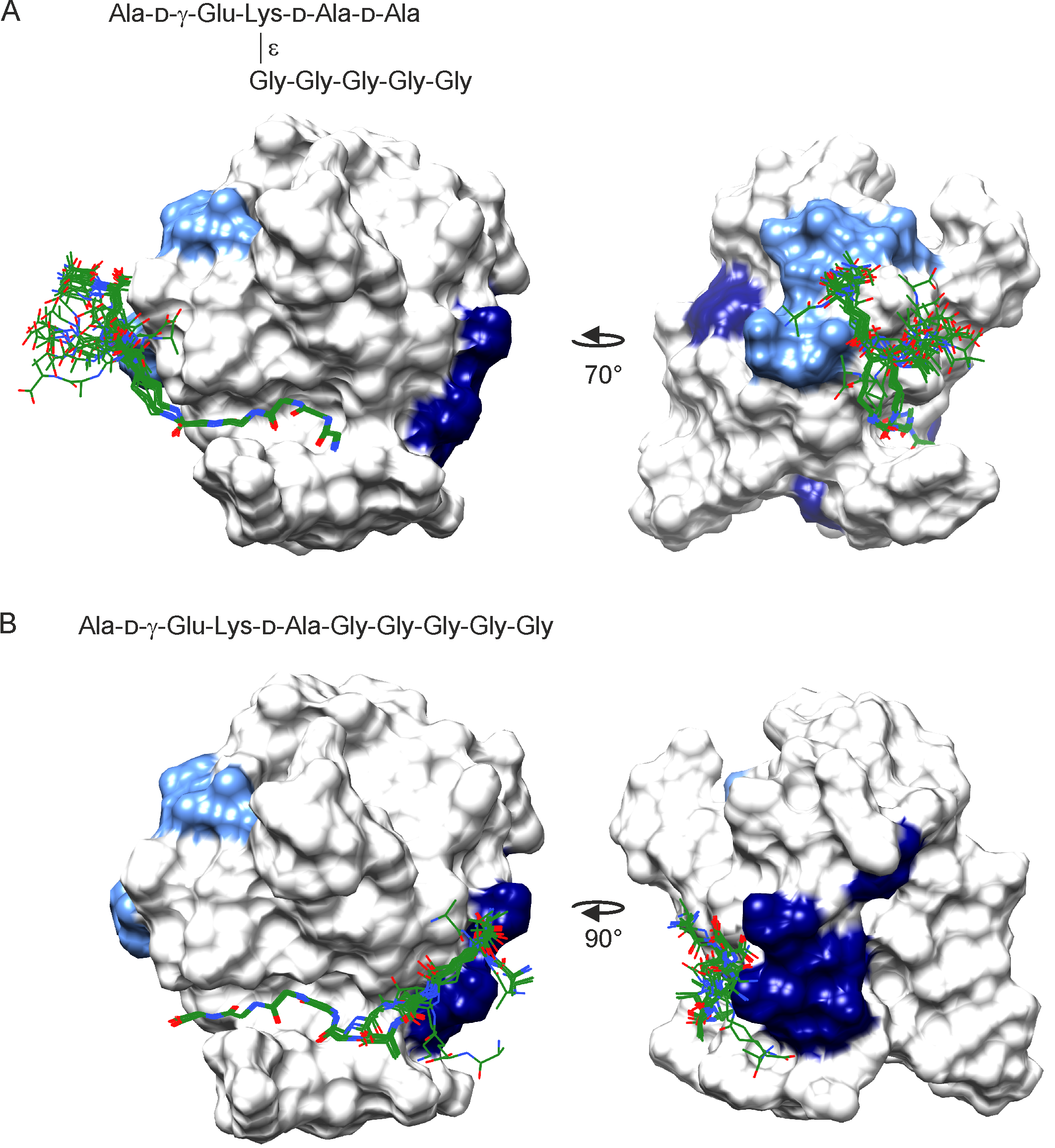

Supplement: Supplementary file 10 [file Image_9.TIF]
